# Feedback between high-pressure genesis of abiotic methane and strain localization in subducted carbonate rocks

**DOI:** 10.1038/s41598-020-66640-3

**Published:** 2020-06-17

**Authors:** Francesco Giuntoli, Alberto Vitale Brovarone, Luca Menegon

**Affiliations:** 1 Institut de Minéralogie, de Physique des Matériaux et de Cosmochimie (IMPMC), Sorbonne Université, Muséum National d’Histoire Naturelle, UMR CNRS 7590, IRD UR206, 75005 Paris, France; 2 Department of Biological, Geological and Environmental Sciences, Università degli Studi di Bologna, via Zamboni 67, 40126, Bologna, Italy; 3 Department of Earth Sciences, University of Torino, Via Valperga Caluso 35, I-10125 Torino, Italy; 4 School of Geography, Earth and Environmental Sciences, University of Plymouth, Plymouth, PL4 8AA UK; 5 The Njord Centre, Department of Geosciences, University of Oslo, P.O. Box 1048 Blindern, Norway

**Keywords:** Structural geology, Petrology, Geochemistry

## Abstract

Fluid-rock interactions exert key control over rock rheology and strain localization. Redox may significantly affect the reaction pathways and, thereby, the mechanical properties of the rock. This effect may become critical in volatile-rich, redox sensitive rocks such as carbonate-rich lithologies, the breakdown of which can significantly modify the net volume change of fluid-mediated reactions. Subduction focus the largest recycling of crustal carbonates and the most intense seismic activity on Earth. Nevertheless, the feedbacks between deep carbon mobilization and deformation remain poorly investigated. We present quantitative microstructural results from natural samples and thermodynamic modeling indicating that percolation of reducing fluids exerts strong control on the mobilization of carbon and on strain localization in subducted carbonate rocks. Fluid-mediated carbonate reduction progressed from discrete domains unaffected by ductile deformation into localized shear zones deforming via diffusion creep, dissolution-precipitation creep and grain boundary sliding. Grain-size reduction and creep cavitation along localized shear zones enhanced fluid-carbonate interactions and fluid channelization. These results indicate that reduction of carbonate rocks can exert an important positive feedback on strain localization and fluid channelization, with potential implications on seismic activity and transport of deep hydrocarbon-bearing fluids.

## Introduction

It is suggested that C cycling at subduction zones can be nearly balanced to 50–300 km depth and over a very short time span of about 5–10 m.y^1^. (for reference, the residence time of carbon in the convective mantle is estimated between 1 and 4 b.y.; e.g.^2^). This means that large amounts of subducted C may be mobilized during metamorphism and transferred back to shallower reservoirs without reaching the convective mantle. This effect is enhanced in the presence of fluid percolation, which promotes metamorphic reactions and dissolution of C-bearing minerals^3,4^.

When devolatilization of subducted C reservoirs happens, also the mechanical properties of the country rock may be affected. However, the relationships between carbon degassing and deformation in subducting slabs remain poorly investigated. In this perspective, forearc depths are relevant as they host the incipient stages of C outgassing from subducted slabs^1,4,5^ and the most extreme manifestations of intermediate seismicity (e.g.^6^). Carbonate minerals represent the largest carbon reservoir being subducted and recycled at convergent margins^7^, and their stability may be strongly affected by redox^8^. As fluid release drastically decreases the strength of a of rock volume (e.g.^9^ and references therein), and the destabilization of carbonate minerals may simultaneously decrease the volume of the rock and increase the amount of free fluid^4,10,11^, redox-controlled mobilization of subducted carbonates^12,13^ may potentially affect the rheology of deep rocks and cause mechanical instabilities at convergent margins.

Carbonate degassing at forearc depth has long been considered to happen at relatively oxidized conditions, and releasing C-bearing fluid species such as CO_2_ or HCO_3_^−^ (e.g.^3,14^). The potential role of CO_2_ release on mechanical weakening includes variations in the wetting properties, water fugacity and related effects on mineral assemblages and porosity^15–17^. These effects seem negligible on quartz and basaltic rocks, as indicated by some experimental laboratory studies^18,19^, but may be different on other systems where CO_2_ is produced in response to, e.g., T-dependent mineralogical reactions^17,20,21^.

On the other hand, deep carbonate degassing under reducing conditions at which methane (CH_4_) is stable, and its implications on deformation have not been investigated to date. Over the last years, a series of studies have shown that such reducing conditions can be achieved in subducting slabs^12,13,22–24^. The interest towards reducing conditions relies on the fact that low *f*O_2_ can enhance carbonate decomposition and carbon mobility with respect to more oxidized conditions^8^.

In this paper, we investigate the feedbacks between carbonate reduction and CH_4_ production, carbon mobility, and deformation through the study of natural samples of carbonate-bearing high-pressure rocks exhumed in the Lanzo Massif, Western Alps. The Lanzo Massif (Supplementary Fig. S1) represents a portion of subcontinental lithospheric mantle that experienced a polyphase evolution from Jurassic extension and exhumation at the Tethys seafloor, to Eocene subduction and high-pressure/low-temperature metamorphism to ~550–600 °C and 2–2.5 GPa, and successive exhumation in the Western Alps metamorphic belt^25–27^. The present-day structure of the Lanzo Massif consists of a large body of fresh peridotite surrounded by a shell of serpentinite that formed as a result of polyphase hydration of the peridotite from the seafloor to subduction and exhumation settings (e.g.^25,28–30^). The serpentinized shell includes bodies of carbonated serpentinites, or ophicarbonates, interpreted to have formed as a result of hydrothermal alteration of the ultramafic body at the seafloor prior to Alpine subduction^25,30^. Part of these carbonated rocks recorded fluid-mediated reduction and associated genesis of abiotic CH_4_ —followed by partial C reprecipitation as graphite— in the Alpine subduction zone, as indicated by mineralogical, fluid inclusion, and stable isotope data^22^. This event was constrained at metamorphic conditions of ~450–350 °C and ~1–0.5 GPa across the aragonite-calcite transition during the exhumation of the Lanzo Massif. This study presents the quantitative microstructural characterization and thermodynamic modelling of samples that were previously used to constrain the petrological and geochemical evolution of the reduction event (Supplementary information).

## Results

### Patterns of fluid-mediated reduction and strain localization

**Figure 1:**
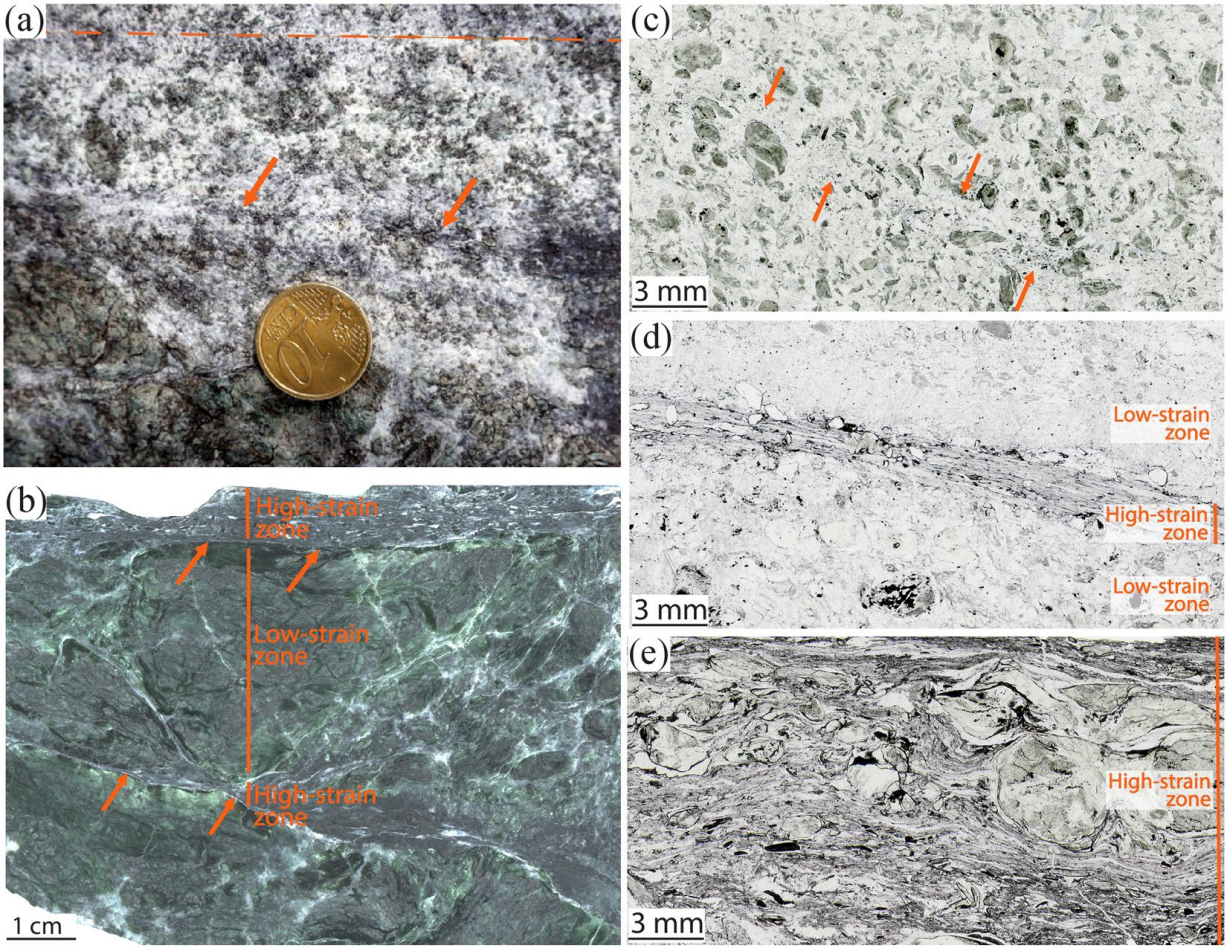
Structural characteristics of the carbonated serpentinite. (**a**) Fresh outcrop with serpentinite clasts in a calcite-rich matrix. The matrix ranges in colour from white to silvery grey, depending on the amount of graphite. Dashed line highlights the weak foliation; arrows indicate a shear zone rich in graphite. (**b**) Hand specimen of carbonated serpentinites cut by shear zones rich in graphite, highlighted by the arrows (sample 5). (**c**–**e**) Thin-section optical microphotos of the three stages of fluid-mediated carbonate reduction structures, marked by the presence and abundance of graphite. Plane‐polarized light. (**c**) Incipient carbonate reduction marked by slight graphite precipitation along a band; sample 15–19 (see also Fig. S2a–c). (**d**) Moderate carbonate reduction with the development of a narrow shear zone highlighted by graphite; sample 15–2 (details in Fig. 2). (**e**) Enhanced carbonate reduction with the development of a wide shear zone rich in graphite wrapping around serpentinite clasts; sample 5 (details in Supplementary Fig. S5).

The carbonated serpentinite consists of rather isotropic Ca-carbonate-rich matrix embedding clasts of serpentinite (Fig. 1). The modal proportions of serpentinite and carbonate in rocks unaffected by reduction processes are strongly variable across the body and mainly depends on the size of the serpentinite clasts, which ranges from sub-mm scale to about 1 m in length (Fig. 1a). Localized, graphite-rich domains can be identified in the field, with a thickness ranging from few millimetres to ~0.5 m and length up to several metres, light to dark grey in colour depending on the graphite abundance (Fig. 1b). Previous stable isotope data of residual carbonate in variously reacted samples indicate that the distribution and amount of graphite in the rock follow the degree of carbonate reduction^22^ (see below for details). The graphite amount was therefore used as a microstructural proxy for the extent of carbonate reduction within from least to most reacted samples (Supplementary Table S1 for bulk stable isotope data). Field and microstructural observations indicate a strong correlation between the distribution of graphite, and thereby the degree of carbonate reduction, and the high-pressure, antigorite-bearing localized shear zones inside the rock. Reduction and ductile deformation increase together from incipient, to moderate, and enhanced. The carbonate-serpentinite ratios typically decrease as a function of the degree of fluid-mediated carbonate reduction, suggesting progressive carbonate consumption with reduction (Supplementary information for discussion on stable isotope data).

Incipient carbonate reduction is represented by isolated, sub-cm networked veins and channels cutting through carbonate-dominated portions of the rock, and are marked by a slight and discontinuous graphite precipitation that can be identified only at the microscale (Fig. 1c and Supplementary Fig. S2). In the country rock, carbonate crystals are equant and up to several mm in size. Pre-kinematic serpentine in serpentinite clasts and isolated crystals (Srp1 hereafter) is represented by antigorite, the high-temperature serpentine polysome stable at depths ≳35 km in the Alpine subduction^31^ (Supplementary Fig. S3). Microstructural and previous stable isotope data for this sample indicate a very slight degree of reduction (Fig. 1c; Supplementary Table S1). No significant ductile deformation was observed along the incipient reduction channels (Fig. 1c, Supplementary Fig. S2), suggesting that the reduction slightly preceded the activation of ductile shear zones.

**Figure 2:**
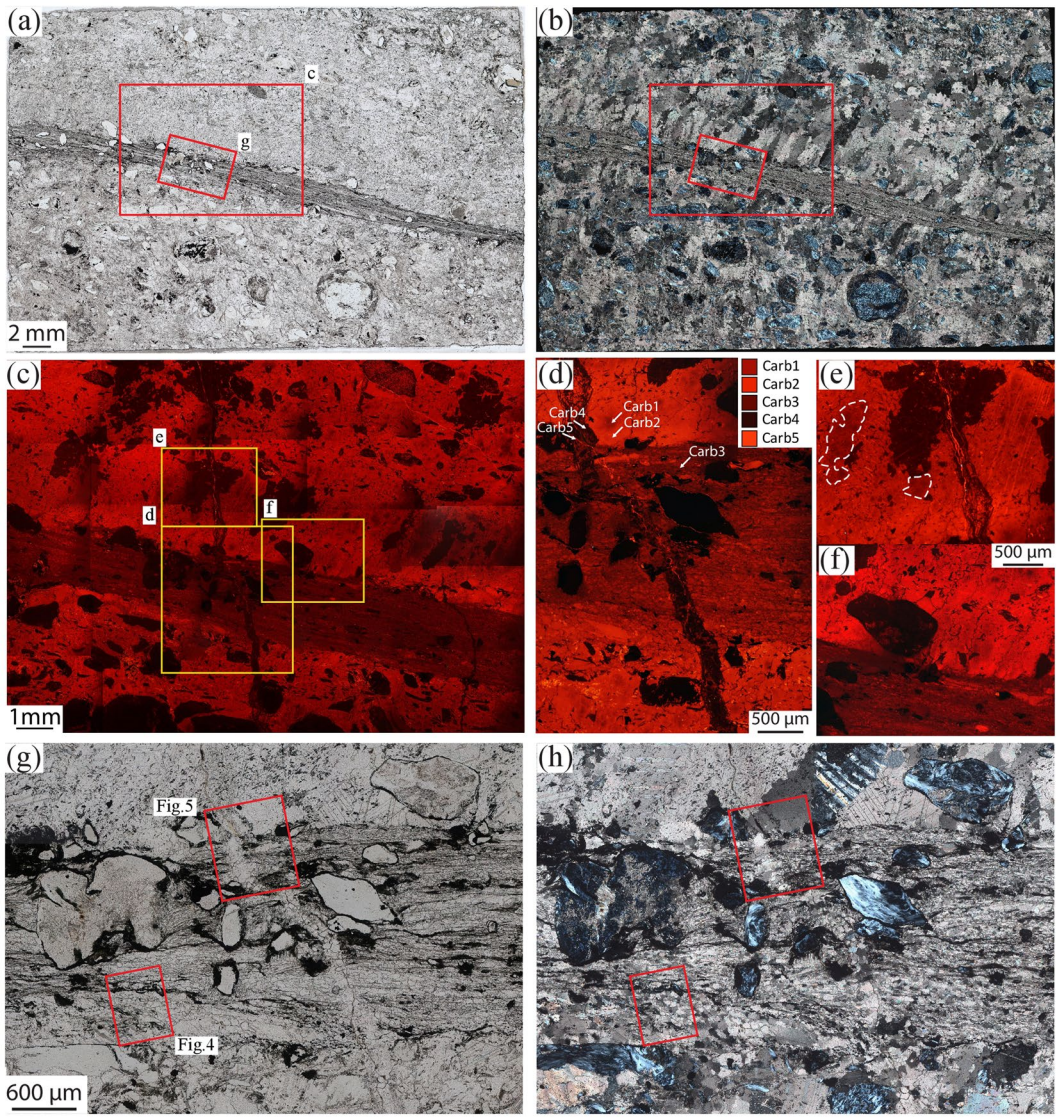
Microstructural features of the carbonated serpentinites. (**a**) Thin-section optical scan of sample 15-2 highlighting the graphite-rich shear zone. Plane‐polarized light. (**b**) The host rock is characterized by serpentinite clasts and calcite crystals some mm in size. At the edges of the shear zone calcite displays a columnar habit; their asymmetry is consistent with a dextral-sense of shear. Crossed‐polarized light. (**c**–**f**) CL images highlighting the different calcite generations in all the structural domains of the sample (note that Carb2 represent pseudomorphs over aragonite, see text for details). The yellow rectangles indicate the location of (**d**,**e**,**f)**. (**d**) Vein system cutting at high angle both the shear zone and the host rock, and sealed by Carb4. Carb5 crystallizes inside the latest fracture. (**e**) Columnar crystals of Carb2 with relic cores of Carb1 (two cores highlighted by the dashed white lines). Note the twin lamellae in both generations, suggesting topotactic replacement of Carb1 by Carb2. (**f**) Columnar Carb2 crystals cut by the shear zone. (**g,h**) Details of (**a**,**b**), respectively. Graphite is more abundant in the shear zone where it rims serpentinite clasts and crystallises along foliation planes, conferring a turbid aspect to it. Note the drastic decrease in the grain size occurring in the shear zone.

**Figure 3:**
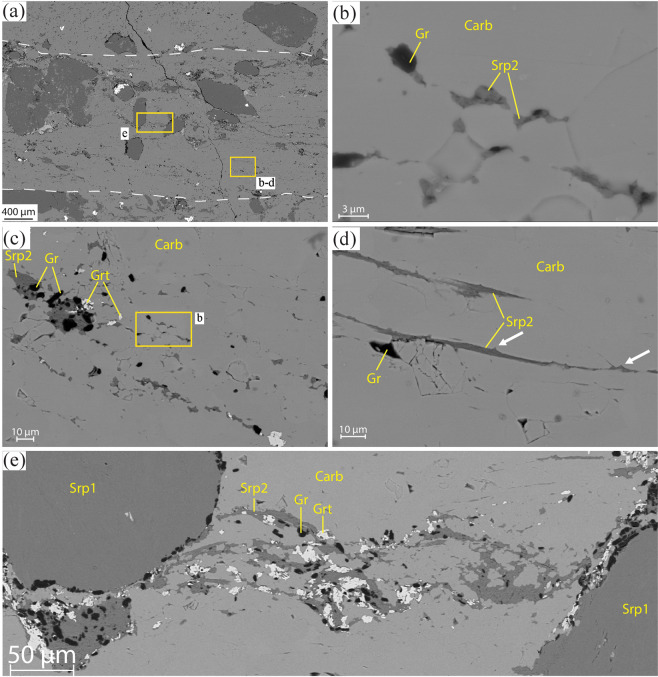
Details of Fig. 2g,h. (**a**) BSE image showing the relations between the shear zone (white dashed lines) and the host-rock. A foliation is visible in the shear zone marked by Srp2 (dark grey), garnet (light grey) and graphite (black). Magnetite crystals (white) occur, especially outside the shear zone. (**b**–**e**) BSE images highlighting the relation between Ca-carbonate (grey), serpentine and graphite in different structural sites. (**b**) Srp2 and graphite occurring at grain boundaries and triple junctions between polygonal calcite grains, interpreted as creep cavities. (**c**) Aligned trails of Srp2, graphite and garnet in a C’-planes orientation (for a dextral sense of shear), interpreted as creep cavitation bands. (**d**) C’-plane marked by Srp2. Note the triangular protrusions representing former grain boundaries (arrows). (**e**) Syn-deformational minerals describing the foliation inside the shear zone between two serpentinite clasts (Srp1).

Moderate reduction is characterized by higher graphite proportions along strongly localized shear zones of about ~2 mm in thickness, which represent the evolution of the incipient reduction channels with increasing reduction (Figs. 1d and 2). Inside the shear zones, carbonate shows a significant and abrupt decrease in grain size from several mm down to few tens of µm, and a shape from equigranular and polygonal to elongated (Figs. 2g,h and 3). Serpentinite clasts proximal to or within the shear zone are coated with graphite aggregates up to 100 µm thick, reflecting an increasing graphite content towards the shear zone. A second generation of syn-kinematic antigorite (Srp2; see Supplementary Table S2 for compositional variations relative to Srp1), newly formed andradite, graphite, and diopside crystallize at carbonate grain boundaries and in the pressure shadows of deformed serpentinite-clasts, with a grain size ranging from few µm to 100 µm (Fig. 3). Antigorite habit along grain boundaries varies from almost equant, ~5 µm long, to acicular, more than 50 µm long and ~5 µm wide. Individual crystals link to form discrete planes, up to several hundreds of µm long (Fig. 3c–e; see location in Fig. 3a). A carbonate vein system cuts the shear zone at high angle, as well as carbonate and serpentinite clasts in the host rock (Fig. 2c–e). The veins are filled with a new Ca-carbonate generation containing longitudinal trails of CH_4_-H_2_-bearing fluid inclusion (Supplementary Fig. S4).

Enhanced carbonate reduction is represented by large, ~30 cm thick, deep-dark domains hosted in a serpentinite-dominated rock (Fig. 1b,e and Supplementary Fig. S5). The reduction in this case is confined along a complex network of graphite-rich shear zones separating carbonate-richer low-strain domains. The low carbonate/serpentinite ratios of these rocks may reflect the enhanced carbonate reduction and concentration of serpentinite clasts. Besides the size and minor mineralogical variations, the microstructural fingerprints of the intensely reduced and deformed domains are overall equivalent to the moderate reduction channels. An exception is the common presence of syn-kinematic carbonate veins with crack-seal texture formed within the shear zones (Supplementary Fig. S2). These veins are visible also in the field with thickness up to some cm and up to several tens of cm in length (Supplementary Fig. S2).

### Microstructural fingerprints of carbonate evolution

Cathodoluminescence (CL), used in conjunction with microstructural analysis, allowed distinguishing different generations of Ca-carbonate based on their luminescence colour, ranging from dark to bright orange in the studied samples. These different generations do not show substantial chemical variations in major elements (Supplementary Table S2), except some minor (~0.1 wt %) MgO variations in samples affected by enhanced reduction (Supplementary Fig. S6).

Five carbonate generations were distinguished from the least to the most reacted samples and are best summarized by samples affected by moderate reduction along discrete shear zones. Complementary information for samples affected by incipient and enhanced reduction are available in the Supplementary information. The first two generations (Carb1 and Carb2) are characteristic of the pre-reduction carbonated serpentinite. Carb1 consists of fractured relict cores up to few mm in size evidenced by a dark red luminescence colour and surrounded by a brighter generation (Carb2; Fig. 2d–f). Carb2 also forms large grains, several mm in size, moderately elongated in the host rock and with a columnar habit at the edges of the shear zone. These two generations do not show any evidence for reduction, such as graphite precipitation, CH_4_-bearing fluid inclusions, or isotopic reset (Supplementary Table S1).

**Figure 4:**
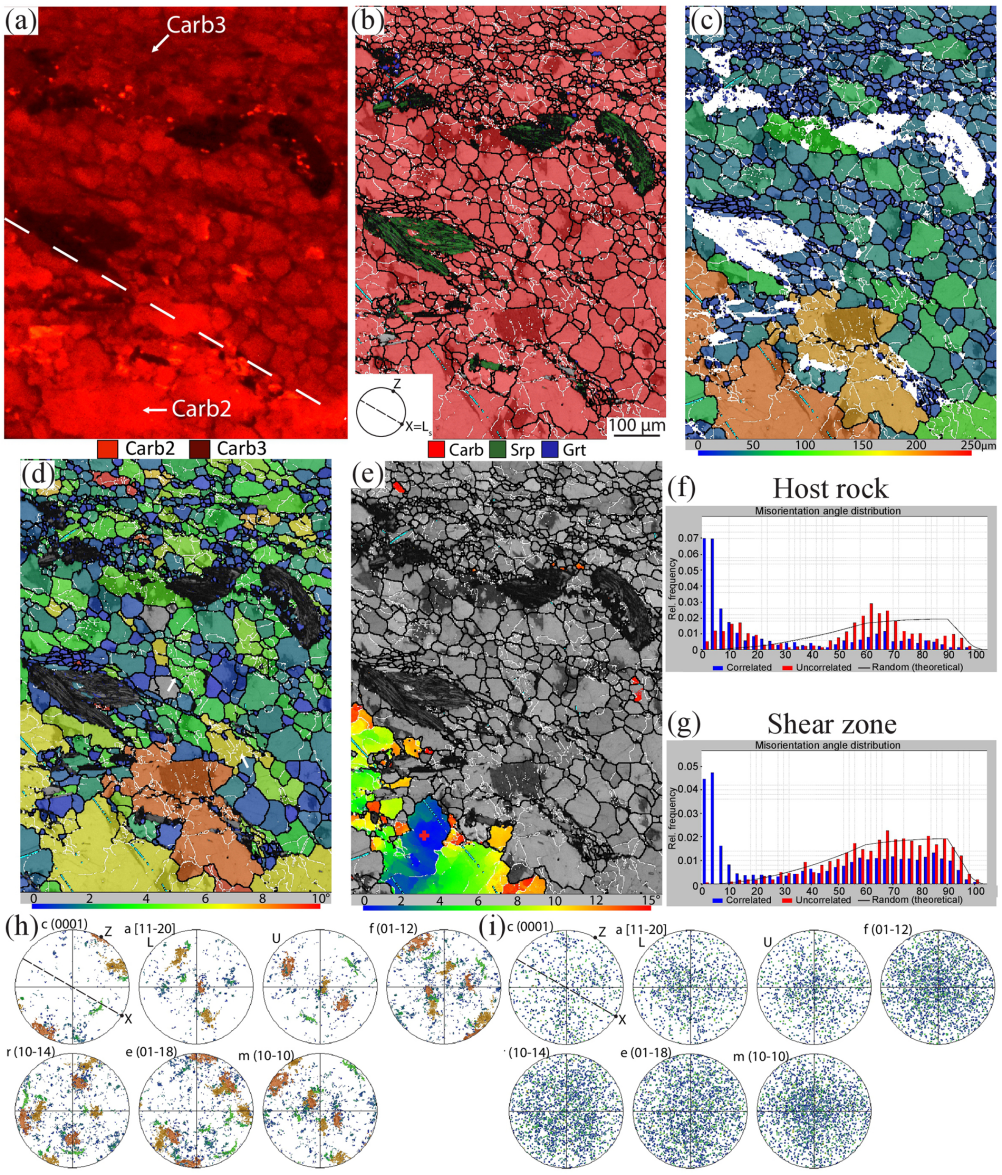
CL image and EBSD map 1 displaying the lower contact between shear zone and host-rock, see location in Fig. 2g. (**a**) CL image highlighting Carb2 and Carb3. The white dashed line highlights the boundary between shear zone (above) and host-rock. (**b**) EBSD phase map. In all the EBSD maps white lines represent low‐angle boundaries (2–10° of misorientation), black lines represent high angle boundaries (misorientation > 10°), light blue lines correspond to twin boundaries in calcite. (**c**) Calcite grain size (GS) map. (**d**) Calcite GOS map. The white arrows indicate subgrains of the same size as the recrystallized grains (see text for discussion). (**e**) Texture Component map (TC map) showing the misorientation from a reference point (marked by a red cross). The misorientation increases from the core to the rim of the crystal, and it correlates with the occurrence of low- and high angle boundaries. (**f**,**g**) Histograms of distribution of misorientation angles for calcite showing the highest peaks for values <10° for correlated pairs. Host rock and shear zone, respectively. (**h**,**i**) pole figures of the crystallographic orientation data of calcite for the host rock and the shear zone, respectively, colour-coded according to the grain size map shown in (**c**). Note the attenuation of CPO that occurs from the host rock to the shear zone. (**h**) 106016 data points; (**i**) 858 data points (one-point-per-grain). X = L_s_ is the stretching lineation, Z is the pole of the shear zone boundary, stereographic projections, L is lower hemisphere, U is upper hemisphere, lower hemisphere if not specified.

**Figure 5:**
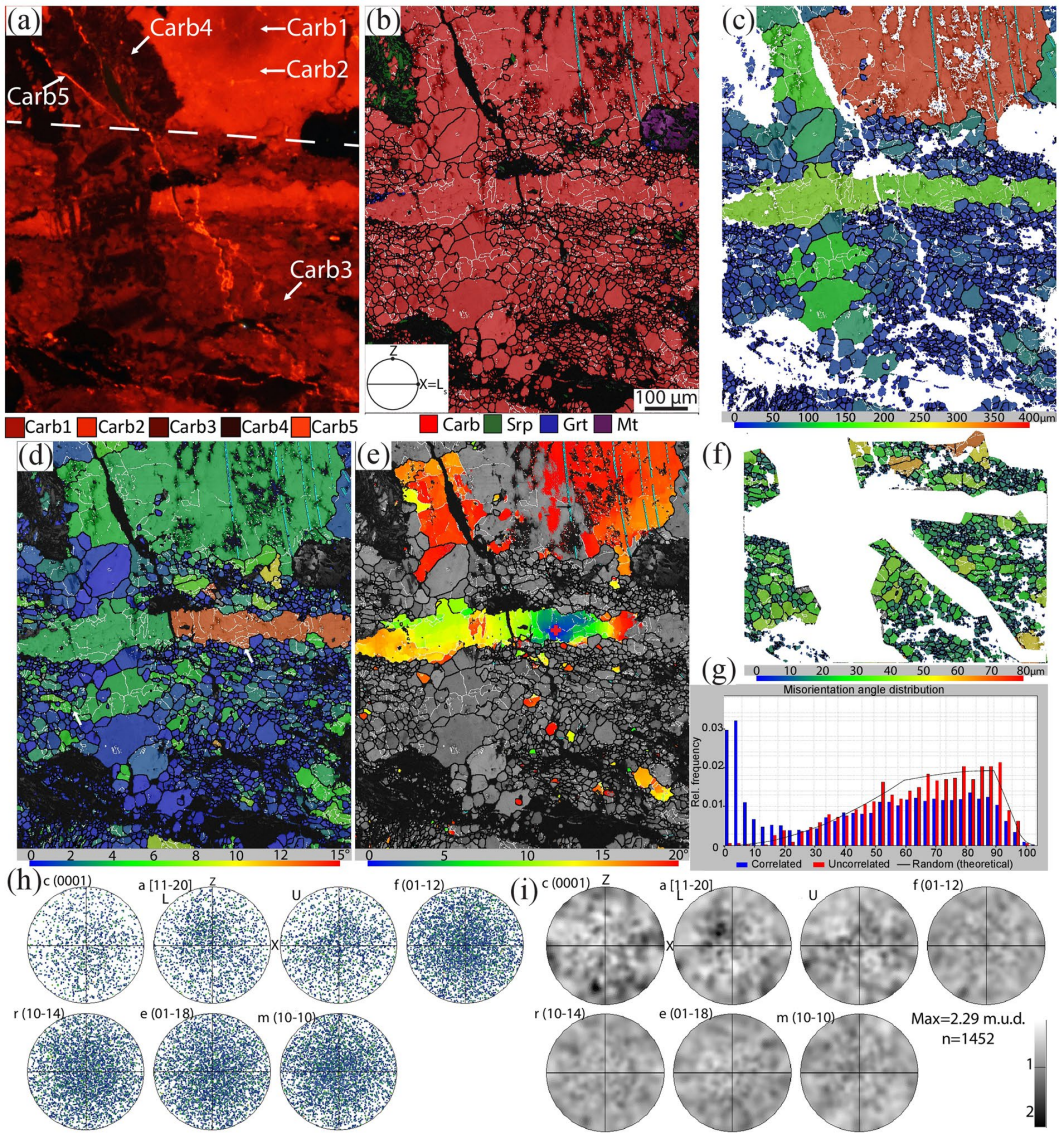
CL image and EBSD map 2 displaying the upper contact between shear zone and host-rock, see location in Fig. 2g. (**a**) CL image highlighting the textural position of all the calcite generations. The white dashed line highlights the boundary between shear zone (below) and host-rock. (**b**) EBSD phase map. (**c**) Calcite GS map. The host rock has calcite crystals with a grain size of several hundred µm, the shear zone with a grain size between few µm and 70 µm, the vein system between 50 and 150 µm. (**d**) Calcite GOS map; the long fragment inside the shear zone displays the highest GOS values (see text for discussion). Carb3 and 4 generations have GOS values generally between 1 and 4°. The white arrows indicate subgrains of the same size as the recrystallized grains. (**e**) TC map showing the misorientation from a reference point (marked with a red cross). The long fragment inside the shear zone shows increasing misorientation values from the core to the rim. The increasing misorientation correlates with the occurrence of low angle boundaries. (**f**) Carb3 GS map (shear zone). (**g**) Histograms of distribution of misorientation angles for Carb3 (shear zone) showing the highest peaks for values <10° for correlated pairs of data points. (**h**,**i**) Pole figures (**h**) and contoured pole figures (**i**) of the crystallographic orientation data of Carb3. Note that no CPO is present in the shear zone. 1452 data points (one-point-per-grain). n = number of grains. Half width 10° and cluster size 5°, maximum value is given.

Carb3 characterizes the shear zone and shows cross-cutting relationships with the previous carbonate generations. It has a darker CL colour than Carb2 and has a grain size of maximum 100 µm (Figs. 4a and 5a). The abundant precipitation of graphite and the stable isotope signature of this generation (Supplementary Table S1) indicate that it marks the first reduction event.

Carb4, the darkest in CL colour, fills fractures that cut across all the previous generations (Figs. 2c–e and 5a). As Carb3, also this generation is associated with H_2_-CH_4_-rich fluid circulation, as indicated by the presence of such species in fluid inclusions within the vein system (Supplementary Fig. S4). Carb5 is the brightest generation in CL colour and appears as a late stage filling small cracks and along grain boundaries (Fig. 5a). This generation will not be discussed further.

### Mechanisms of strain localization and estimate of strain rates

Electron backscattered diffraction (EBSD) analyses were focused along the edges of (map 1 and 2) and inside (map 3; Supplementary information) shear zones of samples characterized by moderate and enhanced reduction. Maps 1 and 2 investigate the drastic decrease of calcite grain size from the host rock to the shear zone: from a maximum of few mm in the former (Carb1–2) to a maximum of 100 µm and an average grain size ~10 µm in the latter (Carb3; Figs. 4 and 5). Calcite displays a further decrease in grain size (between few µm and 30 µm) along shear bands in a C’ orientation in the shear zone, and it has an equigranular/polygonal or slightly elongate shape parallel to the shear zone.

Carb1–2 grains at the edges of the shear zone display rare e-twins (rotation angle of 77° around the 0–221 axis). Carb1-2 grains contain several low angle boundaries, interpreted as subgrain boundaries, and some high angle boundaries, interpreted as grain boundaries (Figs. 4b and 5b). Subgrain boundaries are usually absent in grains smaller than 30 µm. Grains larger than 30 µm have GOS values (i.e., a measure of the internal strain of a grain, see Methods) ranging from 3 to 8°, with maximum values up to 11° (Figs. 4c,d and 5c,d), and contains subgrains of the same size of recrystallized Carb3 grains in the shear zone. Crystals <30 µm display generally GOS values <1°. The exceptions are represented by the crystals located inside the veins (Carb4) that have GOS values <1° even though they have a grain size up to 200 µm, suggesting that these grew after the shear zone (map 2, Fig. 5d; more details in the following).

The bigger columnar grains located at the edges of the shear zone share a weak crystallographic preferred orientation (CPO), with the (0001) maxima perpendicular to the shear zone boundary (Fig. 4h); this CPO is absent or less defined in the shear zone (Figs. 4i and 5h,i). The misorientation angle distribution for correlated pairs of data points in carbonate grains outside the shear zone displays the strongest peaks between 2 and 10°. Misorientations ≤ 15° between uncorrelated pairs also occur more frequently than for the theoretical random distribution. Overall, the misorientation angle distribution is consistent with the presence of subgrain boundaries^32^ (Figs. 4f,g and 5g). Based on the presented data, in the moderate reduction sample the columnar carbonate crystals located at the edges of the shear zone (Carb2) grew perpendicularly and oriented (i.e., with a CPO) inside pre-reduction fractures (e.g.^33^). These columnar crystals most likely represent pseudomorphs over aragonite, based on their morphology (e.g.^34^) and on the presence of aragonite crystals still preserved as inclusions in magnetite and garnet in the studied rocks^22^ (Supplementary Fig. S4).

In the shear zone, Carb1-2 crystals experienced grain size reduction by progressive subgrain rotation recrystallization that developed new smaller and more equant grains (Carb3) at the expenses of the big columnar ones (Figs. 4b, 5b and Supplementary Fig. S7^34,35^;). These new grains were further deformed by diffusion creep and grain boundary sliding, as indicated by the attenuation and eventual loss of the CPO^36–38^ (Fig. 4h,i and Supplementary Fig. S7). This deformation mechanism favored the nucleation of new syn-deformational phases along Carb3 grain boundaries (e.g. abundant graphite, Srp2, andradite ± diopside; Fig. 3b), probably as a consequence of creep cavitation (as defined by^39–41^). These creep cavities eventually linked together and formed creep cavitation bands parallel to C and C’-planes by grain boundary sliding (Fig. 3c–e). Creep cavities and creep cavitation bands were sealed by syn-deformational phases, most commonly Spr2. Furthermore, phase nucleation during creep cavitation enhanced phase mixing and the associated pinning effects, thus preventing grain growth of Carb3 grains and further localizing the deformation along discrete bands in the shear zone. EBSD map 3 show similar deformation microstructures, which are also indicative of the additional contribution of dissolution–precipitation creep (Supplementary information).

Additionally, inside the shear zone, large crystals with higher values of GOS and abundant low angle boundaries can be observed, as in map 2 (Fig. 5). CL patterns highlight that this crystal is Carb2 (bright in CL), the left side of the same crystal is instead Carb4 (dark colour in CL; Fig. 5a). This difference in CL is not visible from the crystallographic orientation data. The same observation is valid also in the host rock in the big crystal located at the top of map 2 cut by the vein system, and in general for all the calcite crystals located in the host rock cut by such vein system (Fig. 2c–e). These data suggest dissolution of Carb2 and pseudomorphic and topotaxial growth of Carb4, as the latter shares the same orientation of the parental crystal^42–45^. Thus, dissolution-precipitation processes occurred along such conduits and in their proximity (few hundreds of µm away).

**Figure 6:**
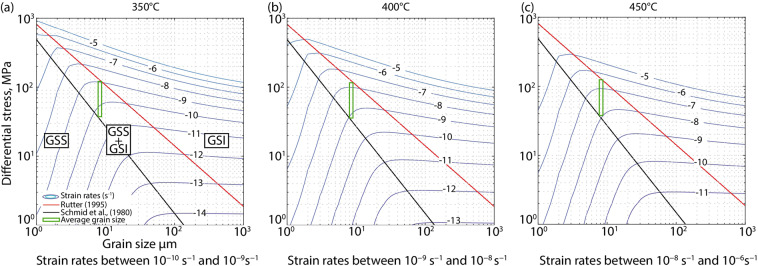
Deformation mechanism map for calcite for 350, 400 and 450 °C respectively. GSS and GSI: grain size sensitive and insensitive creep, respectively.

Figure 6 shows the deformation mechanism maps for calcite for the temperature range 350–450 °C (Methods). We note that in all the EBSD maps, relict grains (characterized by GOS values typically >2°) contain subgrains of the same size as the recrystallized grains (with GOS values < 2°), indicating that dynamic recrystallization of calcite occurred by subgrain rotation and that the grain size of recrystallized grains was not modified after recrystallization. Thus, the recrystallized grain size piezometer for calcite can be used to estimate the flow stress at the time of dynamic recrystallization in the shear zone. The grain size piezometers^46,47^ suggest differential stresses between 70 and 150 MPa for an average grain size of 8.8 µm (Fig. 6 and Supplementary Fig. S8). We used the calcite flow laws for grain-size insensitive dislocation creep^48^ and for grain-size sensitive creep involving diffusion and grain-boundary sliding^49^ to estimate the strain rates in the shear zone. We obtained strain rates in the range of 10^−9^ s^−1^ to 10^−6^ s^−1^, with the faster strain rates found for the higher temperature bound (Fig. 6).

### Fluid-rock redox evolution during fluid infiltration

**Figure 7:**
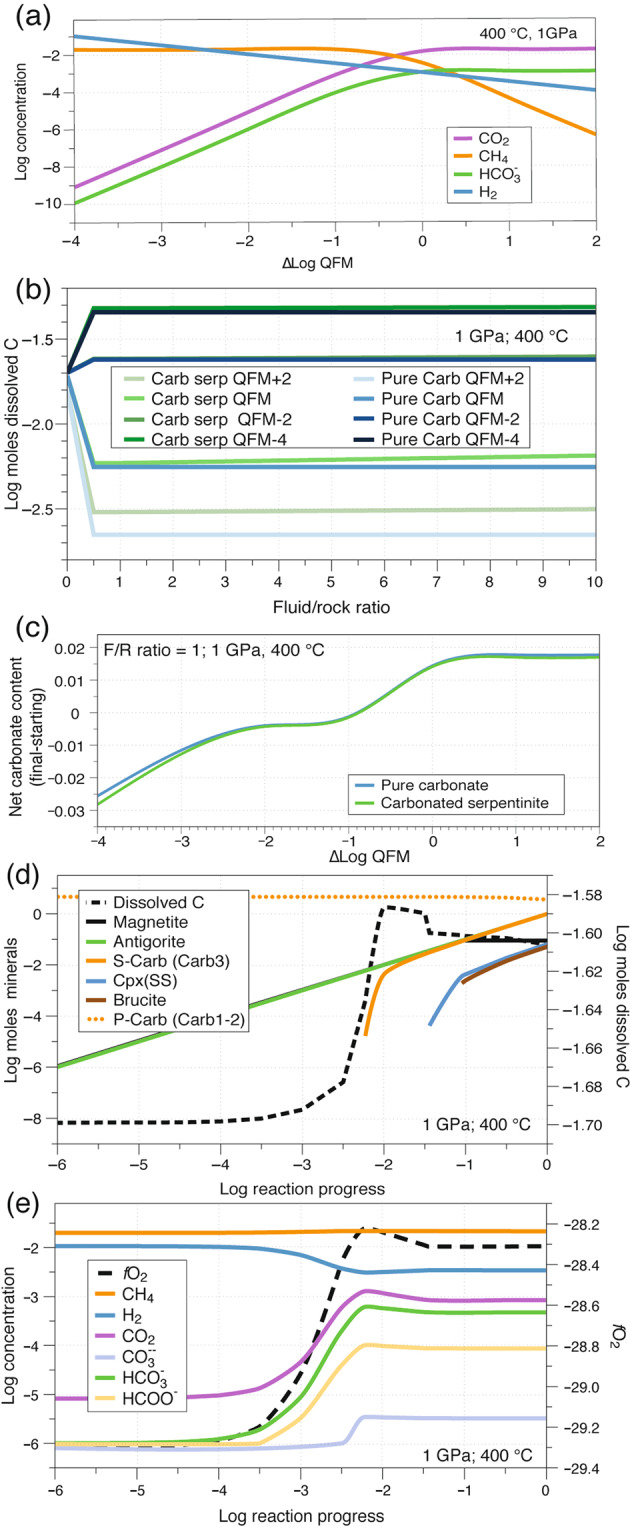
Thermodynamic modelling results. (**a**) Selected fluid component speciation of a fluid in equilibrium with a serpentinite at 400 C° and 1 GPa, as function of the *f*O_2_ (as ∆Log QFM). (**b**) Total dissolved carbon during infiltration of external fluids into a carbonated serpentinite and into a pure carbonate rock. The redox state of the infiltrating fluids is shown as ∆Log units relative to the QFM buffer in the legend. (**c**) Net carbonate budget (final-starting, in moles) at reaction completion as function of the *f*O_2_ of the infiltrating fluid. The plotted data are for fluid/rock ratios equal to unity; higher fluid/rock ratios do not substantially affect the results. (**d**) Mineralogical evolution for a fluid at ∆QFM = −2 interacting with a carbonated serpentinite and fluid/rock ratios equal to unity. The molality of total dissolved carbon is also displayed. P-Carb: primary carbonate, chemically equivalent to Carb1-2 in the studied rocks. S-Carb: secondary carbonate, approaching the composition of Carb3 in the studied samples and all figures. (**e**) Selected fluid component speciation during the fluid/rock interaction of panel (d). The evolution of the *f*O_2_ during the reaction progress is also displayed. For reference, the *f*O_2_ at QFM at 400 °C and 1 GPa is −27.3.

The presented microstructural analysis of variably reduced and deformed samples indicates a strong correlation between carbonate reduction and strain localization, which suggests the potential role of reducing fluids to weaken subducted carbonate-rich rocks and localize deformation. In particular, the observed deformation mechanism of dissolution-precipitation creep requires the presence of a fluid phase to activate dissolution-precipitation processes along the reaction channels. In order to test the potential for reducing fluids to activate and sustain these deformation mechanisms, and finally to postulate feedback between carbonate reduction and deformation, we performed thermodynamic modeling with the Deep Earth Water (DEW) model^50,51^ (Methods; Fig. 7). In the models, a carbonated serpentinite was reacted with fluids equilibrated with a serpentinite at redox conditions from +2, to −4 log units relative to the quartz-fayalite-magnetite (QFM) buffer, and for fluid/rock ratios from 0.5 to 10 (Fig. 7a; Methods). An additional set of calculations were done with a pure carbonate rock as a reacting rock in order to model fluid infiltration along serpentine-free portions of the rock. All models were calculated at 400 °C and 1 GPa.

The results show that the redox state of the infiltrating fluid strongly controls the extent of net carbonate dissolution, with total dissolved C at reaction completion being more than one log unit higher for reduced infiltrating fluids than oxidized ones (Fig. 7b). Slight variations in dissolved C concentrations between carbonated serpentinites and pure carbonate are related to silicate-carbonate reactions in the former lithology. The fluid/rock ratio, here used as a proxy for the magnitude of fluid infiltration, appears to have a negligible effect on carbonate dissolution for values higher than 0.5 (Fig. 7b).

The modeling results show that the net carbonate content in the rock at reaction completion (initial minus final carbonate content) is negative for reduced infiltrating fluids and positive for oxidized fluids (Fig. 7c). The threshold between negative and positive net carbonate budget of the interaction, which is outlined by the change in slope of the curves in Fig. 7c, roughly corresponds to the condition of equal molality of H_2_ and CO_2_ and CH_4_ > CO_2_ in the infiltrating fluid, corresponding to about ∆Log QFM = −0.7 at 400 °C and 1 GPa (Fig. 7a).

Results for both carbonated serpentinite and pure carbonate show a net carbonate volume decrease of about 2.5% for infiltrating fluids buffered at ∆Log QFM = −4 (Fig. 7c). More oxidized infiltrating fluids (>QFM) define a plateau of net carbonate volume increase of about 2%. The modeled reaction pathway for infiltration of reducing fluids (Fig. 7d) highlights the progressive consumption of the initial carbonate (compositionally equivalent to Carb1-2 in the natural samples), followed by reprecipitation of a new generation of carbonate towards equilibration of the system. The modeled secondary carbonate shows a slight enrichment in MgO compared to the primary carbonate. This compositional variation matches the composition of syn-kinematic Carb3 in samples affected by enhanced reduction and deformation (Supplementary Figs. S6, S7). Secondary antigorite, magnetite, diopside and brucite also form during the fluid/rock equilibration (Fig. 7d), as also observed in the natural samples in creep cavities and cavitation bands (Supplementary Fig. S6; see also^22^).

During reaction progress, the *f*O_2_ of the reacting system increases as a result of the release of oxygen from carbonate consumption, with a final slight decrease related to the precipitation of secondary carbonate (Fig. 7e). The *f*O_2_ at reaction completion is higher than the infiltrating fluid, but still reduced relative to the QFM buffer (for reference, the *f*O_2_ at QFM at 400 °C and 1 GPa is −27.3). Over the entire reaction pathway, CH_4_ and H_2_ appear as dominant fluid species over CO_2_. The model also shows that the CH_4_ concentration in the fluid slightly increased towards reaction completion owing to the hydrogenation of CO_2_ released by carbonate dissolution.

## Discussion

**Figure 8:**
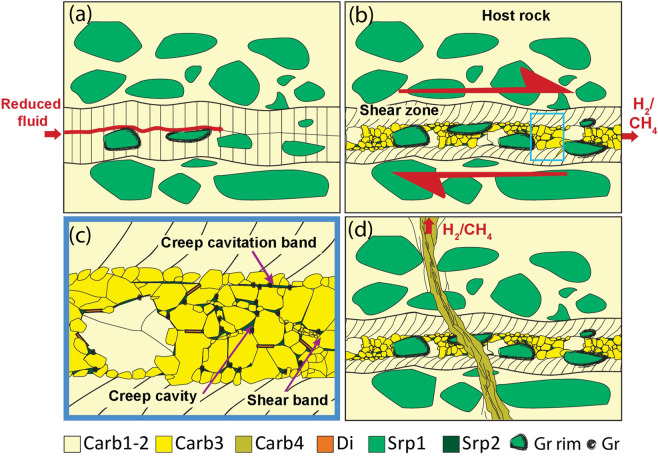
Summary sketch of the interactions between H_2_-CH_4_-rich fluids, deformation and metamorphism in the carbonated serpentinites. (**a**) Infiltration of reducing fluids started. In this sketch, based on the moderately reduced sample (Fig. 1), the infiltration proceeded along a pre-existing discontinuity represented by columnar carbonate. Incipient reduction of Carb_2_ and local precipitation of graphite are also displayed, marking the onset of carbonate dissolution-precipitation. (**b**) Grain size reduction of the carbonate crystals occurred by subgrain rotation recrystallization (Carb3). Subsequently, diffusion creep and grain boundary sliding resulted in a progressive loss of carbonate CPO. (**c**) Detail of selected area. Srp2, graphite and diopside grew in creep cavities and pressure shadows, promoting phase mixing, pinning the grain size inside the shear zone and favouring the localization of the deformation. Deformation continued producing a dextral shear zone. Creep cavities coalesced developing creep cavitation bands (C’-planes) rich in Srp2, graphite, and diopside. Partial Carb3 dissolution and topotactic precipitation occurred. (**d**) A vein system cut all the previous structures. Along this vein system, inclusions of CH_4_ and H_2_ are found. Carb4 sealed these veins, with partial pseudomorphic topotaxial replacement of the previous Ca-carbonate generations.

The collected data suggest that fluid-mediated reduction of carbonate rocks can favour strain localization at pressure and temperature conditions corresponding to the forearc region of subducted slabs. In particular, no significant ductile deformation was observed along the incipient reduction channels, and thermodynamic modeling results highlighted that the infiltration of reduced fluids in carbonate rocks promotes dissolution-precipitation processes, which appears to be key to strain localization in the studied carbonate rocks. Figure 8 summarises the proposed evolution of the studied shear zones based on the presented microstructural and thermodynamic data, with particular reference to the microstructures observed in the moderately reduced sample (Fig. 2). Infiltration of reducing fluids started along pre-existing discontinuities (e.g.^52^) such as carbonate veins, and along carbonate-serpentine interfaces (Fig. 8a). With increasing reduction, carbonate dissolution-precipitation processes started, as predicted by the thermodynamic models, with higher dissolution rates for intensely deformed crystals characterized by higher strain energy^53^. Thus, syn-kinematic reduction played a fundamental role on the development of the observed shear zones, as evidenced in the natural samples by microstructural features characteristic of dissolution-precipitation creep (Supplementary Fig. S7).

Grain size reduction of carbonate crystals occurred by subgrain rotation recrystallization. Deformation proceeded with diffusion creep and grain boundary sliding, which resulted in the progressive weakening of the carbonate CPO (Fig. 8b). Additional secondary minerals such as Srp2 and graphite grew in dilatant sites such as creep cavities and pressure shadows, promoting phase mixing and pinning the grain size inside the shear zone, which further facilitated the localization of deformation via grain size sensitive creep (Fig. 8c). The contribution of both grain size sensitive and insensitive creep is also supported by the deformation mechanism maps for calcite, where the recrystallized grain size of our sample plots at the transition between these two fields (Fig. 6). Deformation continued with the formation of mature shear zones and the development of shear bands in a C’ orientation that contain the same mineral phases that grew in dilatant sites. Creep cavities eventually coalesced to produce creep cavitation bands, mostly sealed by Srp2. Syn-kinematic carbonate veins observed in the most reacted/deformed samples suggest that veining might have occurred syn-ductile deformation (Supplementary Fig. S2). Summarizing, these processes resulted in a dynamic evolution of grain size, mineral assemblage, porosity and fluid flow in a localized brittle-viscous shear zone.

Finally, a post-kinematic vein system cut all the previous structures at high angle (Fig. 8d). The carbonate filling these vein (Carb4) partially replaced pseudomorphically and topotaxially all the previous carbonate generations. The post-shear zone CH_4_-H_2_-bearing Carb4 veins suggest that the reducing fluids may have been episodically pumped-out from these domains. These brittle structures may have formed as a result of brittle failure by local fluid overpressure during progressive carbonate reduction and CH_4_ generation, as suggested by the abundance of CH_4_-rich fluid inclusions along these structures (Supplementary Fig. S4).

The thermodynamic models confirm that, despite the release of oxygen in the fluid during carbonate reduction, the fluid remained reduced relative to the QFM buffer and CH_4_-H_2_-rich (Fig. 7e). For fluid/rock ratios as low as 0.5 (and higher) —for reference, time-integrated fluid fluxes in lithospheric channelized fluid conduits are generally >10^4^ m^3^_(fluid)_/m^2^_(rock)_^54^—, infiltration of reducing fluids (<QFM) is capable to trigger and maintain CH_4_ generation and associated dissolution-precipitation mechanisms controlling deformation. Thus, this mechanism can impart a strong positive feedback between reduction and CH_4_ generation, and strain localization in carbonate rocks.

The development of new discontinuities, such as shear zones, also favors fluid channelization^55^, which may sustain protracted carbonate reduction, deformation and CH_4_ migration potentially over large distances, as observed in similar geological contexts for aqueous fluids^56^. However, fluid flowing into and being channelized within a ductile shear zone requires local dilatancy in the shear zones itself, because the mean pressure within a weak ductile shear zone is higher than the surrounding matrix, so that fluids will tend to be expelled from the shear zone^57^. In our samples, deformation by grain size sensitive creep in the fluid rich shear zone reflects a marked weakening of the shear zone compared to its surrounding matrix. Creep cavities and creep cavitation bands filled with graphite and with Serp2 represent the signature of the dynamic porosity generated and maintained during viscous creep in order to channelize fluid flow in the shear zone. Moreover, isolated fluid-filled porosity in the shear zone will be close to lithostatic pressure, so that the hydrofracture criterion could locally be reached at the grain scale and along the grain boundaries. Continuous C’ planes defined by Srp2, as well as protrusions of Srp2 along Carb-Carb grain boundaries (Fig. 3d) and syn-kinematic veins (Supplementary Fig. S2) may reflect local hydrofracturing in the shear zone.

Relative to reduced fluids, infiltration of more oxidized aqueous fluids would result in much less net carbonate dissolution (Fig. 7) and, plausibly, in a less prominent effect on dissolution creep and strain localization. Moreover, when CO_2_ is thermodynamically favored over CH_4_, the process of carbonate dissolution is inhibited to proceed further^8^, thus limiting the positive feedback on carbon mobility in deep fluids. Additionally, fluids containing more abundant CO_2_ over CH_4_ (for *f*O_2_ ≳ QFM, Fig. 7a) would also precipitate more carbonate relative to CH_4_-dominated fluids (*f*O_2_ < QFM, Fig. 7c). In metamorphic systems controlled by decarbonation reactions, e.g., T-dependent carbonate-silicate reactions releasing CO_2_, structural discontinuities may also form, as indicated by experimental results^17^. In particular, it has been shown that quartz-carbonate (+H_2_O) assemblages reacting in an imposed thermal gradient to form wollastonite + CO_2_ can develop porosity channels parallel to the gradient^17^, formation of which may promote localization of fluids and deformation. Nevertheless, the same study showed that this process is strongly dependent on T gradients and on the presence of mineral assemblages reacting to release carbon (e.g. quartz + carbonate) rather than on the redox state of the system. In the absence of such conditions (e.g., in the carbonate + H_2_O system), the formation of porosity channels was shown to be weak^17^. These features suggest that, relative to reducing conditions, oxidizing conditions limit the positive feedbacks between fluid infiltration, net carbonate dissolution, and carbon mobility, which appear to have played a significant role on the development of localized deformation in the studied carbonate rocks. The effect of *f*O_2_ on deformation of rocks free of significant volatilization potential, such as dry silicate rocks, may be different^58^.

The presented data show that fluid-mediated carbonate reduction favoured strain localization at metamorphic conditions equivalent to about 35 km depth, which also correspond to the depths of intense seismic manifestations at convergent margins (e.g.^59^). This, together with the increasing reports of reduction processes in subduction zone rocks^22–24^, raises questions about the potential role of fluid mediated carbonate reduction on the genesis of seismic activity in subduction zones. The estimated differential stresses and strain rates are consistent with previous results on carbonate mylonites/ultramylonites^36,60^, and overall indicative of fast deformation events that are likely to be related to the earthquake cycle (e.g.^61,62^). This is also confirmed by the documented meso and microstructural features of the studied shear zones, which are consistent with deformation mechanisms analogous to those expected to reflect seismic activity, such as ductile deformation and coeval carbonate veins with crack-seal textures, creep cavitation bands and fractures (e.g.^63^). Additionally, the described microstructures can be upscaled to the mesoscale, where shear zones and coeval veins with crack-seal textures are present with a length up to several metres (Supplementary Fig. S2).

Concluding, this study provides the first evidence that fluid-mediated reduction of subducted carbonate rocks and associated genesis of abiotic CH_4_-rich fluids can promote strain localization and potentially seismic activity at convergent margins. These structures not only maintain a positive feedback between CH_4_ genesis and deformation, but could also act as preferential fluid pathways for the migration of deep unconventional hydrocarbons from their source regions towards shallower reservoirs.

## Methods

### Optical cathodoluminescence (CL)

Optical cathodoluminescence (CL) was performed at the Sorbonne Université, Paris (FR), using an Opéa cold-cathode at 15 kV, ~300 μA/mm^2^ under a pressure of ~0.01 Bar, coupled with a Nikon optical microscope and a Nikon D70 camera.

### Scanning electron microscopy (SEM) and electron backscatter diffraction (EBSD)

Scanning electron microscopy (SEM) was performed on carbon-coated thin sections using a Zeiss Ultra 55 field emission gun at the Sorbonne Université. Analyses were conducted under high vacuum, using an accelerating voltage of 15 kV and working distance of 7.5 mm. Backscattered electron (BSE) analyses were performed using an Angle Selective Backscattered Detector or an Energy Selective Backscattered Detector.

Electron backscattered diffraction (EBSD) analyses were performed with a Jeol JSM 6610LV SEM at the Electron Microscopy Centre of the University of Plymouth (UK). EBSD patterns were collected with 20 kV accelerating voltage, 18–23 mm working distance, 1 µm step size and a 70° sample tilt. AZtec software (Oxford Instruments) was used to automatically index diffraction patterns. Successively, raw maps were processed with HKL Channel 5 (Oxford Instruments), performing the noise reduction procedure tested by^64^. Grains smaller than 3 times the step size were excluded from the dataset. The mean angular deviation values were 0.47–0.58 for calcite, the raw indexing rate ranged between 76% and 91%, mainly due to poor indexing of serpentine. Crystallographic orientation data were plotted on pole figures (stereographic projection; upper and lower hemispheres), with X parallel to the stretching lineation and Z parallel to the pole of the mylonitic foliation. The grain orientation spread maps (GOS maps) were used to display the intensity of internal strain of individual grains. GOS is defined as the average misorientation angle between each pixel in a grain and that grain’s average orientation.

### Electron probe micro-analyser (EPMA)

EPMA analyses were performed at the Camparis Platform, Sorbonne Université, using a Cameca SX Five connected to five spectrometers. Both spot analyses and X-ray maps were acquired with wavelength dispersive spectrometers (WDS). Firstly, spot analyses were acquired for each mineral phase, successively X-ray maps were collected on overlapping areas. Spot analyses were acquired with 15 KeV accelerating voltage, 10 nA specimen current and ~1 µm beam diameter. The following standards were used to measure ten oxide compositions: orthoclase (Al_2_O_3_, K_2_O), garnet (SiO_2_, MgO, FeO), albite (Na_2_O), diopside (CaO), manganese titanate (TiO_2_, MnO), chromium oxide (Cr_2_O_3_). X-ray maps were acquired with 15 KeV accelerating voltage, 10–100 nA specimen current, dwell times of 100 ms and step size of 1 µm. Ten elements (Si, Ti, Al, Fe, Mn, Mg, Na, Ca, K and Cr) were collected in two series for intensity X-ray maps. Successively, intensity X-ray maps were processed using XMapTools 2.6^65^, using spot analyses as internal standard to obtain concentration maps of oxide weight percentage.

### Raman spectroscopy

Raman spectra were obtained using a Renishaw InVIA Reflex microspectrometer at the Laboratoire de Géologie of the Ecole Normale Supérieure, Paris, France using a 514 nm laser in polarized mode delivering 20 mW on the sample, a long-working-distance ×50 Leica objective lens with 0.5 numerical aperture, and 1800 grooves/mm gratings. Measurements were done on polished thin sections. The spectrometer was calibrated with silicon standard. Acquisition time was 5–10 s, with 2–4 accumulations per spot.

### Estimate of differential stresses and strain rates in the shear zones

Carb3 grain size was used to estimate differential stresses and strain rates responsible for the shear zone formation (Fig. 6). Map 2 offered the best example, as less Carb2 relic grains are present (which contain subgrains of the same size of the recrystallized grains). To eliminate the contribution of such relic grains, we excluded the grains with GOS values > 2°. This value was chosen because grains with GOS < 2° do not contain subgrains and are interpreted to be recrystallised grains. The average grain size is 8.8 µm (Supplementary Fig. S8). Equivalent calculations for aragonite, which was initially stable during reduction^22^ could not be performed, owing to the lack of grain size piezometers for this mineral. Nevertheless, considering that (1) previous work on these samples indicates that the reduction event protracted through the aragonite-calcite transition into the stability of calcite^22^, and (2) the results obtained using the flow laws for calcite and aragonite are comparable^66^, our estimates on calcite result appropriate.

### Thermodynamic modelling

Thermodynamic calculations were done with the Deep Earth Water (DEW) model^50,51^ and the EQ. 3/EQ. 6 software^67^ with a modified Berman thermodynamic database^68^. Firstly, we calculated with EQ. 3 the composition of a fluid in equilibrium with a serpentinite assemblage consisting of antigorite + magnetite + brucite + chlorite + olivine, which represents the most likely source of reducing fluids in the considered case study (Vitale Brovarone *et al*., 2017). The *f*O_2_ of the equilibrium was set at different values corresponding to +2, 0, −2 and −4 log units relative to the QFM (quartz-fayalite-magnetite) buffer. The molality of carbon in the infiltrating fluid was set at 0.02, which represents the threshold for carbonate saturation in the starting serpentinite at 400 °C and 1 GPa. EQ. 6 was then used to model the interaction between the EQ. 3 fluid and a carbonated serpentinite consisting of antigorite (49 vol.%), Ca-carbonate (49 vol.%), and magnetite (2 vol.%) and representing the general composition of the studied rocks prior to reduction. The composition of the main solid solutions in the EQ. 3 and EQ. 6 calculations were set based on the mineral composition analyzed in the samples. An additional set of EQ. 6 calculations were done with the same infiltrating fluid, and a pure carbonate rock composition in order to test the effect of reduction in other carbonate rocks and gave equivalent. Different fluid/rock ratios were considered, from 0.5 to 10, by modifying the number of moles of reactant phases in the calculations.

## Supplementary information

Supplementary Information.

## Data Availability

All data generated or analysed during this study are included in this published article (and its Supplementary Information files).
