# Supplementary material for: Feedback between high-pressure genesis of abiotic methane and strain localization in subducted carbonate rocks
Source: Sci Rep. 2020 Jun 17;10:9848. doi: 10.1038/s41598-020-66640-3 (PMC7300122; doi:10.1038/s41598-020-66640-3)
Supplement: Supplementary file 1 — Supplementary Information. [file 41598_2020_66640_MOESM1_ESM.docx]

# Supplementary information

**Feedback between high-pressure genesis of abiotic methane and strain localization in subducted carbonate rocks**

Francesco Giuntoli ^1,2*^, Alberto Vitale Brovarone ^1,3^, Luca Menegon ^4,5^

^1^ Institut de Minéralogie, de Physique des Matériaux et de Cosmochimie UMR 7590 CNRS-UPMC-IRD-MNHN, Campus Jussieu, Case courrier 115, 4 Place Jussieu, 75005 Paris, France.

^2^ Department of Biological, Geological and Environmental Sciences, Università degli Studi di Bologna, Bologna, Italy

^3^ Department of Earth Sciences, University of Torino, Via Valperga Caluso 35, I-10125 Torino, Italy.

^4^ School of Geography, Earth and Environmental Sciences, University of Plymouth, Plymouth PL4 8AA, UK

^5^ The Njord Centre, Department of Geosciences, University of Oslo, P.O. Box 1048 Blindern, Norway

* Corresponding author.

E-mail address: [francesco.giuntoli@gmail.com](mailto:francesco.giuntoli@gmail.com) (F. Giuntoli)

**
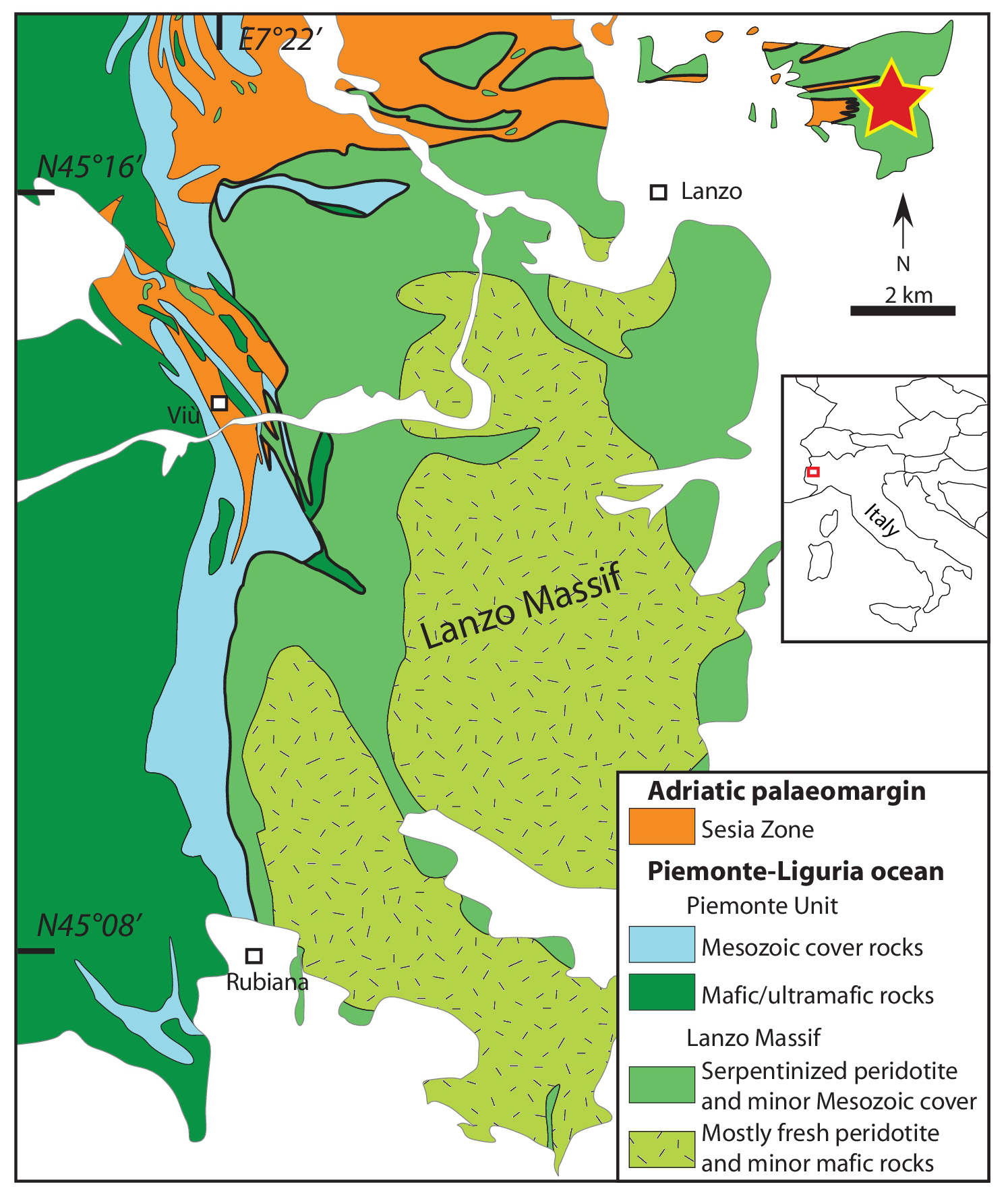
**

**Figure S1.** Geological map of the Lanzo Massif, Italian Western Alps. The star indicates the outcrop location (GPS coordinates: 45°17'46.9"N 7°30'32.8"E), the red square the approximate geological map location (modified from ^1^). Figure created with Adobe Illustrator CS6 (https://www.adobe.com/products/illustrator.html).

## Stable isotopes method and description

One sample (15-2) was analyzed in order to complete the dataset available in ^1^ discussed in the present study. The shear zone of sample 15-2 was sampled by breaking a 2mm slab and successively hand-picking dark fragments corresponding to the graphite-enriched zone of interest. The carbonate concentration and isotopic composition of calcite (δ^13^C) were measured by AP2003 continuous flow isotope ratio mass spectrometer at IPGP, Paris. Between 2 and 3mg of samples were loaded in vials; three standards of pure calcite were also used for calibration of both concentration and isotopic composition. After flushing with ultrapure Helium, orthophosphoric acid (H_3_PO_4_) was introduced in each tube to produce gaseous CO_2_. After 4 h of reaction at ambient temperature, calcite was completely transformed into CO_2_; gases were then transferred into mass spectrometer for analysis. To improve the precision of the measurements, each analysis was repeated four times for each vial; and each sample analysed twice. The isotopic ^13^C/^12^C ratios are expressed using the conventional d-notation versus PeeDee Belemnite (PDB) international standard. The precision is 0.1% for δ^13^C, and 10% for the carbonate content.

The results for sample 15-2 are provided in Table S1 together with previous data from ^1^ discussed in this work. In sample 15-19 the effect of reduction on the bulk-rock carbonate δ^13^C signature is minimal (δ^13^C 2.8‰ reported versus PDB, Table S1), as the rock still falls within the range of marine carbonate values in carbonated serpentinites from various oceanic suites (-6/+3‰, ^2^). In sample 15-2 the localized graphite-bearing channel shows a marked increase in δ^13^C compared to the previous sample, up to 6.8‰, which is indicative of a pronounced reduction in favour of δ^13^C-depleted products such as CH_4_ and/or graphite. In sample 5 the graphite-rich portions in this case has carbonate δ^13^C of 8‰, which is indicative of a higher degree of reduction compared to the previous samples.

| **Sample** | **Type** | **Ductile deformation** | **δ^13^C (‰ vs. PDB)** | **SD (n. 4)** |
| --- | --- | --- | --- | --- |
| **15-19*** | Incipient reduction | None | 2.8 | 0.10 |
| **15-2** | Moderate reduction | Shear zone (2mm thick) | 6.84 | 0.07 |
| **5*** | Enhanced reduction | Shear zone (>30cm thick) | 8.0 | 0.12 |

**Table S1**. δ^13^C of variably reduced ophicarbonates reported versus PDB standard. *From ^1^.


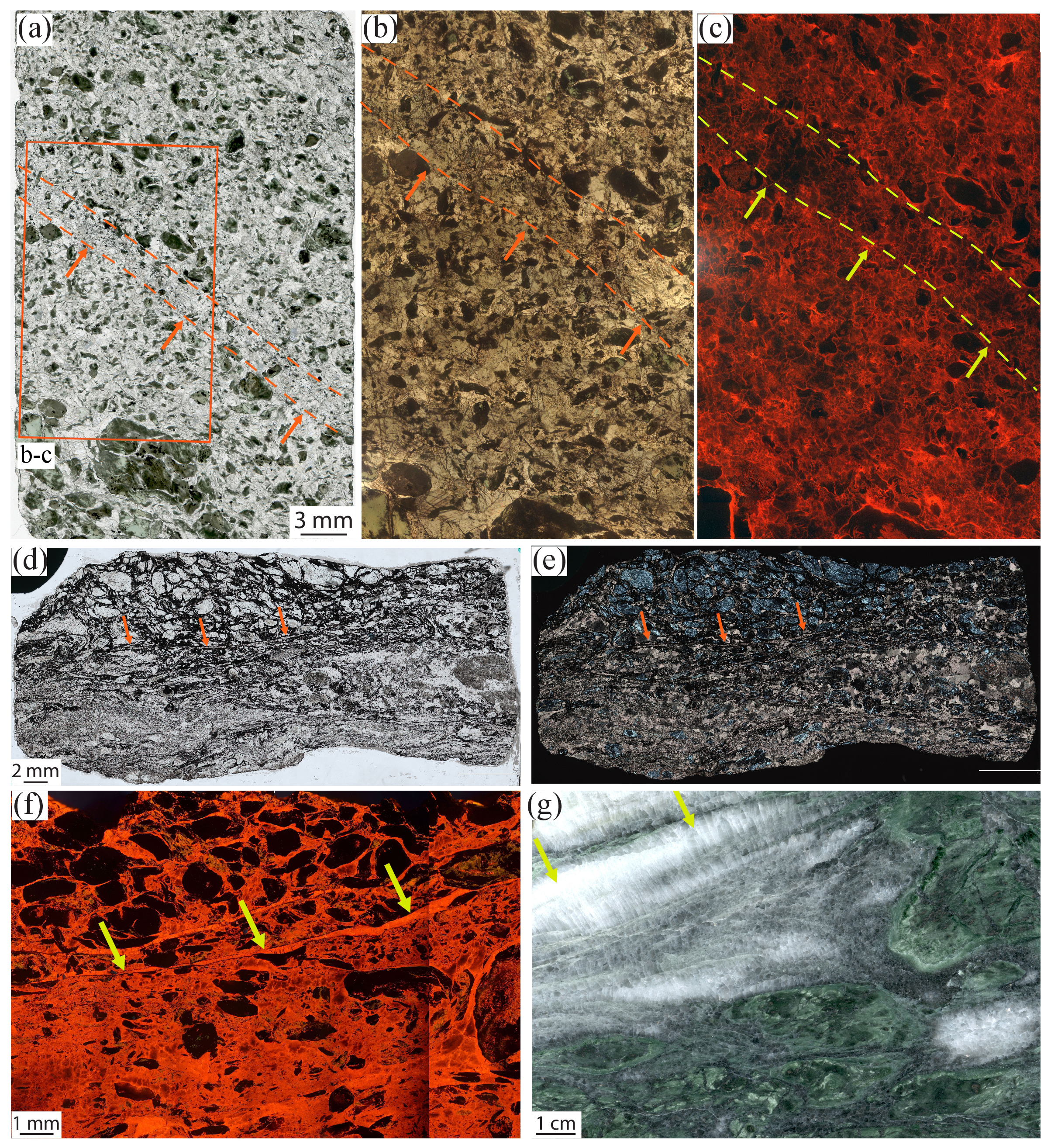


**Figure S2.** Microstructural features of the incipient (sample 15-19; a-c) and enhanced carbonate reduction (sample 5b; d-f). a-b) Carbonated serpentinite with a band highlighted by a slight graphite content. Thin section scan and thin-section optical microphoto and plane‐polarized light, respectively (note that the thickness of this thin section is 100 µm). c) CL image; note the dark carbonate cores surrounded by brighter carbonate with a columnar shape along the channel. d-e) Shear zone with serpentinite clasts rimmed by graphite and graphite-rich shear bands. Note the syn-kinematic carbonate veins with a crack-seal texture parallel to C’-planes indicating a sinistral sense of shear (arrows). Thin-section optical microphotos, plane‐polarized light and crossed‐polarized light, respectively. f) CL image of a different thin section of the same hand-specimen of d and e highlighting the syn-kinematic carbonate veins. g) Hand specimen of carbonated serpentinite displaying syn-kinematic carbonate veins > 5cm.

|  | **Garnet** | | **Serpentine** | | | | **Chlorite** | | **Diopside** | | **Carbonate** | | | | | | | | | | | **Magnetite** | |
| --- | --- | --- | --- | --- | --- | --- | --- | --- | --- | --- | --- | --- | --- | --- | --- | --- | --- | --- | --- | --- | --- | --- | --- |
| **Sample** | **Bal15-2** | | **Bal15-2** | | **1Bal18-2a** | | **Bal15-2** | **1Bal18-2a** | **Bal15-2** | **1Bal18-2a** | **Bal15-2** | | | | | | **1Bal18-2a** | | | | | **Bal15-2** | **1Bal18-2a** |
| **Repr. Spot Analyses** | **CORE** | **RIM** | **Srp1** | **Srp2** | **Srp1** | **Srp2** |  |  |  |  | **Average Carb1-4** | ***SD*** | **Spot Carb1** | **Spot Carb2** | **Spot Carb3** | **Spot Carb4** | **Spot Carb2** | **Average Carb3a** | ***SD*** | **Average Carb3b** | ***SD*** |  |  |
|  |  |  |  |  |  |  |  |  |  |  |  |  |  |  |  |  |  |  |  |  |  |  |  |
| SiO_2_ | 34.92 | 34.72 | 42.73 | 41.50 | 41.27 | 41.94 | 34.37 | 34.58 | 54.76 | 54.40 | - | **-** | **-** | **-** | **-** | **-** | **-** | **-** |  | **-** |  | 0.00 | 0.02 |
| TiO_2_ | 0.85 | 0.05 | 0.03 | 0.01 | 0.01 | 0.01 | 0.01 | 0.00 | 0.03 | 0.05 | - | **-** | **-** | **-** | **-** | **-** | **-** | **-** |  | **-** |  | 0.29 | 0.10 |
| Al_2_O_3_ | 0.55 | 1.13 | 2.59 | 0.36 | 3.38 | 2.08 | 12.45 | 11.88 | 0.10 | 0.15 | - | **-** | **-** | **-** | **-** | **-** | **-** | **-** |  | **-** |  | 0.03 | 0.02 |
| Cr_2_O_3_ | 0.79 | 0.04 | 0.19 | 0.06 | 0.06 | 0.01 | 0.17 | 0.22 | 0.02 | 0.01 | - | - | - | - | - | - | - | - |  | - |  | 0.46 | 0.05 |
| FeO | 25.56 | 26.58 | 2.60 | 3.30 | 4.31 | 5.23 | 3.51 | 4.36 | 1.68 | 2.63 | 0.04 | 0.03 | 0.01 | 0.08 | 0.00 | 0.02 | 0.00 | 0.03 | 0.01 | 0.04 | 0.01 | 91.22 | 90.20 |
| MnO | 0.03 | 0.01 | 0.11 | 0.10 | 0.11 | 0.15 | 0.01 | 0.04 | 0.07 | 0.14 | 0.03 | 0.03 | 0.06 | 0.01 | 0.06 | 0.01 | 0.02 | 0.02 | 0.01 | 0.02 | 0.01 | 0.27 | 0.03 |
| MgO | 0.46 | 0.12 | 39.38 | 38.44 | 37.11 | 37.13 | 35.31 | 34.66 | 17.51 | 17.10 | 0.04 | 0.02 | 0.02 | 0.05 | 0.04 | 0.07 | 0.09 | 0.07 | 0.02 | 0.13 | 0.02 | 0.53 | 0.21 |
| CaO | 34.24 | 34.62 | 0.04 | 0.35 | 0.25 | 0.04 | 0.29 | 0.36 | 25.38 | 25.09 | 55.52 | 1.43 | 55.64 | 55.65 | 55.74 | 55.61 | 55.77 | 55.80 | 1.24 | 55.76 | 1.13 | 0.22 | 0.96 |
| Na_2_O | **-** | **-** | **-** | **-** | **-** | **-** | **-** | **-** | 0.44 | 0.33 | - | **-** | **-** | - | - | - | - | - | **-** | - | - | 0.06 | 0.01 |
| K_2_O | **-** | **-** | **-** | **-** | **-** | **-** | **-** | **-** | **-** | **-** | **-** | **-** | **-** | - | - | - | - | - | **-** | - | - | 0.00 | 0.02 |
| CO_2_ | **-** | **-** | **-** | **-** | **-** | **-** | **-** | **-** | **-** | **-** | 43.65 | 0.52 | 43.74 | 43.78 | 43.83 | 43.73 | 43.88 | 43.90 | 0.41 | 43.94 | 0.50 | - | - |
| Total | 97.41 | 97.27 | 87.69 | 84.12 | 86.49 | 86.59 | 86.11 | 86.11 | 100.00 | 99.91 | 99.27 | - | 99.48 | 99.57 | 99.69 | 99.42 | 99.76 | 99.82 | - | 99.89 | - | 93.10 | 91.62 |
| **Formulae based on 12 O** | | | **on 7 anhydrous O** | | | | **on 14 anhydrous O** | | **on 6 O** | |  |  |  |  |  |  |  |  |  |  |  |  |  |
| Si | 2.92 | 2.90 | 1.98 | 2.01 | 1.95 | 1.99 | 3.27 | 3.31 | 1.99 | 1.99 |  |  |  |  |  |  |  |  |  |  |  |  |  |
| Ti | 0.05 | 0.00 | 0.00 | 0.00 | 0.00 | 0.00 | 0.00 | 0.00 | 0.00 | 0.00 |  |  |  |  |  |  |  |  |  |  |  |  |  |
| Al | 0.05 | 0.11 | 0.14 | 0.02 | 0.19 | 0.12 | 1.40 | 1.34 | 0.00 | 0.01 |  |  |  |  |  |  |  |  |  |  |  |  |  |
| Cr | 0.05 | 0.00 | 0.01 | 0.00 | 0.00 | 0.00 | 0.01 | 0.02 | 0.00 | 0.00 |  |  |  |  |  |  |  |  |  |  |  |  |  |
| Fe^3+^ | 1.79 | 1.86 | **-** | **-** | **-** | **-** | 0.00 | 0.00 | **-** | **-** |  |  |  |  |  |  |  |  |  |  |  |  |  |
| Fe^2+^ | 0.00 | 0.00 | 0.10 | 0.13 | 0.17 | 0.21 | 0.30 | 0.36 | 0.05 | 0.08 |  |  |  |  |  |  |  |  |  |  |  |  |  |
| Mn | 0.00 | 0.00 | 0.00 | 0.00 | 0.00 | 0.01 | 0.00 | 0.00 | 0.00 | 0.00 |  |  |  |  |  |  |  |  |  |  |  |  |  |
| Mg | 0.06 | 0.02 | 2.72 | 2.78 | 2.62 | 2.63 | 5.01 | 4.94 | 0.95 | 0.93 |  |  |  |  |  |  |  |  |  |  |  |  |  |
| Ca | 3.07 | 3.10 | 0.00 | 0.02 | 0.01 | 0.00 | 0.03 | 0.04 | 0.99 | 0.98 |  |  |  |  |  |  |  |  |  |  |  |  |  |
| Na | **-** | **-** | **-** | **-** | **-** | **-** | **-** | **-** | 0.03 | 0.02 |  |  |  |  |  |  |  |  |  |  |  |  |  |
| K | **-** | **-** | **-** | **-** | **-** | **-** | **-** | **-** | **-** | **-** |  |  |  |  |  |  |  |  |  |  |  |  |  |
| ∑ cations | 8.00 | 8.00 | 4.95 | 4.97 | 4.95 | 4.95 | 10.02 | 10.01 | 3.99 | 3.99 |  |  |  |  |  |  |  |  |  |  |  |  |  |

**Table S2**. Representative spot and average composition analyses (wt%) of the mineral phases.

## Petrographic, microstructural and CL supplementary description

The variably reacted carbonated serpentinites are composed of serpentine, Ca-carbonate, chlorite, diopside, and magnetite as dominant pre-reduction assemblage, together with graphite, additional diopside and andraditic garnet in the reduced domains (Supplementary Table S2). Serpentine is antigorite, as indicated by Raman spectroscopy (Supplementary Fig. S3). Ca-carbonate includes both matrix calcite resulting from re-equilibration of the high-pressure polymorph aragonite, and fresh aragonite preserved as inclusion in minerals such as magnetite and garnet (Supplementary Fig. S4). Aragonite+graphite inclusions in magnetite indicate that the reduction process started within the aragonite stability field and continued during exhumation in the calcite stability field. For simplicity, in the following the term carbonate will be used to refer to both calcite and aragonite.

The graphite has a nodular habit and systematically forms along the edges of antigorite crystals or clasts in contact with carbonate, or along fractures inside them. Except for the least reacted sample 15-19, the investigated samples are all characterized by the presence of graphite-rich shear zone, ranging from discrete (few millimetres of thickness; sample 15-2) to pervasive (>30 centimetres of thickness; sample 5), that also display different mineralogical assemblages compared to the host rock.

In sample 15-2 the host rock displays equant and up to several mm in size carbonate crystals. Pre-kinematic antigorite crystals and antigorite serpentinite clasts (Srp1) exhibit scattered and discontinuous graphite rims compared to the shear zone, where the graphite is homogeneously distributed along the carbonate-serpentine interfaces (Fig. 2g, h). At the edges of the shear zone, carbonate fibres up to several mm in size are present and oriented at high angle to the shear zone (Fig. 2b). These carbonate fibres define crack-seal like structures that were also observed in the sample affected by incipient reduction, and structurally pre-date and are variably reworked by both the fluid-mediated reduction and the shear zone. Their asymmetry suggests a dextral sense of shear. In the shear zone, syn-kinematic Srp2 intergrows with andradite and graphite in the pressure shadow of Srp1 clasts (Fig. 3e). Serpentine 1 (Srp1) has higher Al_2_O_3_ content (4.5-2 wt%) than Srp2 (2-0.2 wt%, Supplementary Fig. S6 and Table S2).

Carb3 generation is slightly more complex in the more intensely reduced sample 5, where two sub-generations can be distinguished: Carb3a, dark in CL colour and forming the core of columnar Ca-carbonate inside the shear zone, and Carb3b, corresponding to the 3rd generation in sample 15-2, bright in CL colour and forming along C -planes, grain boundaries of generations 3a, in pressure shadows and fractures (Supplementary Figs. S6d and S7a).


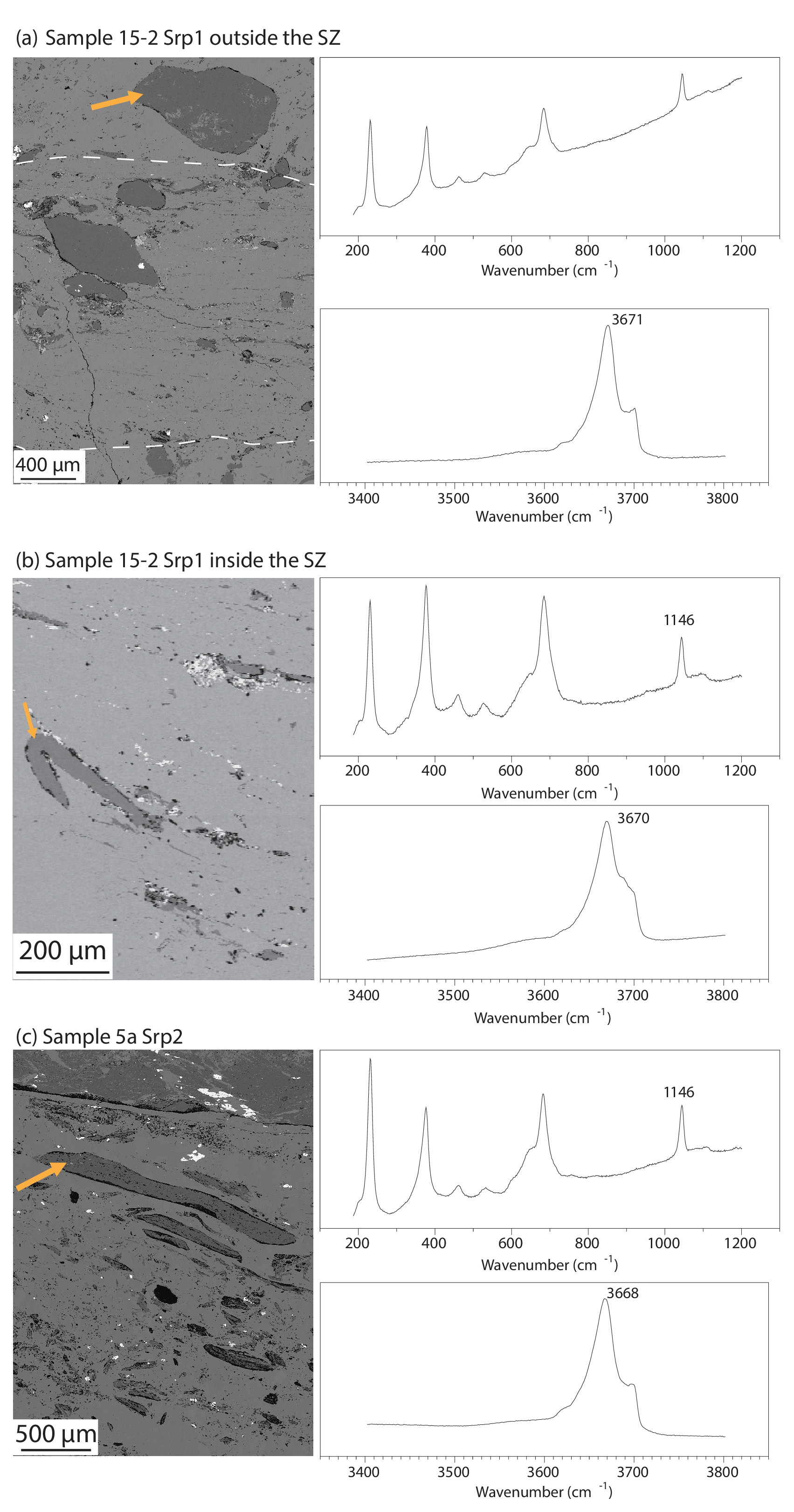


**Figure S3.** BSE images and Raman spectra of representative antigorite crystals. (a) Srp1 located outside the shear zone (white dashed lines); compare with Figs. 2 and 3. (b) Deformed Srp1 located inside the shear zone. (c) Srp2 located inside the shear zone. Compare with Fig. S6.


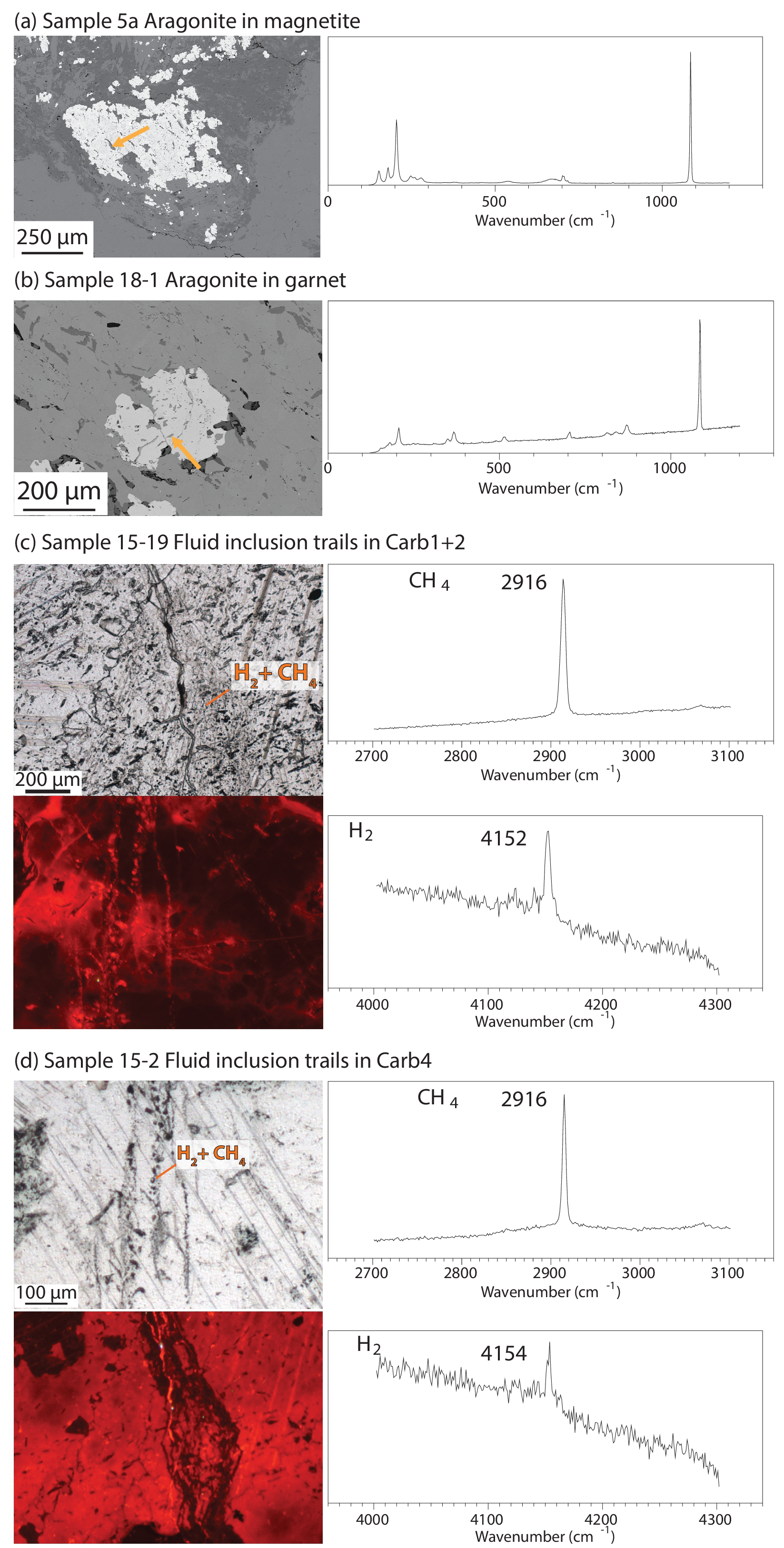


**Figure S4.** Raman spectra of representative aragonite crystals and fluid inclusion trails. (a) Aragonite included in magnetite; BSE image. Position on a larger BSE image in Fig. S5c. (b) Aragonite included in garnet; BSE image. (c-d) CH_4_ and H_2_ fluid inclusion trails. Thin-section optical microphoto with plane‐polarized light and CL image, respectively. Compare (d) with Fig. 2d, e.

## EBSD supplementary analysis

EBSD map 3 shows prismatic calcite grains inside the shear zone (Carb3a in CL), some hundreds of µm in size, cut by a shear band in a C’ orientation along which calcite has a grain size <80 µm (average ~20 µm, Carb3b in CL; Supplementary Fig. S7). The columnar grains contain several low angle boundaries, interpreted as subgrain boundaries, while the smaller calcite grains along shear band are generally free of those. GOS maps are consistent with those shown for the previous sample: bigger crystals have higher GOS values (up to 6°), smaller crystals have lower GOS values (Supplementary Fig. S7d). Few exceptions are present along the shear band, where few grains have GOS values up to 6°. The texture component map shows increasing misorientation from the core to the rim of the columnar crystals (Supplementary Fig. S7e). Pole figures display a well-defined CPO for the columnar subset, with the (0001) maxima coincident with Z (Supplementary Fig. S7h, i). This CPO is less defined for the smaller grains along the shear band (Supplementary Fig. S7l, m). The misorientation angle distribution of calcite displays the strongest peaks between 2 and 10° and minor peaks between 24 and 40°, with higher values for the correlated pairs (Supplementary Fig. S7f, g). Therefore, dissolution and precipitation processes acted in the composite shear zone, with partial dissolution of Carb3a and topotaxial growth of Carb3b (close to the shear band, Supplementary Fig. S7). These processes occurred syn-deformation, as testified by the asymmetry of the Ca-carbonate crystals suggesting dissolution–precipitation creep ^3,4^.

**
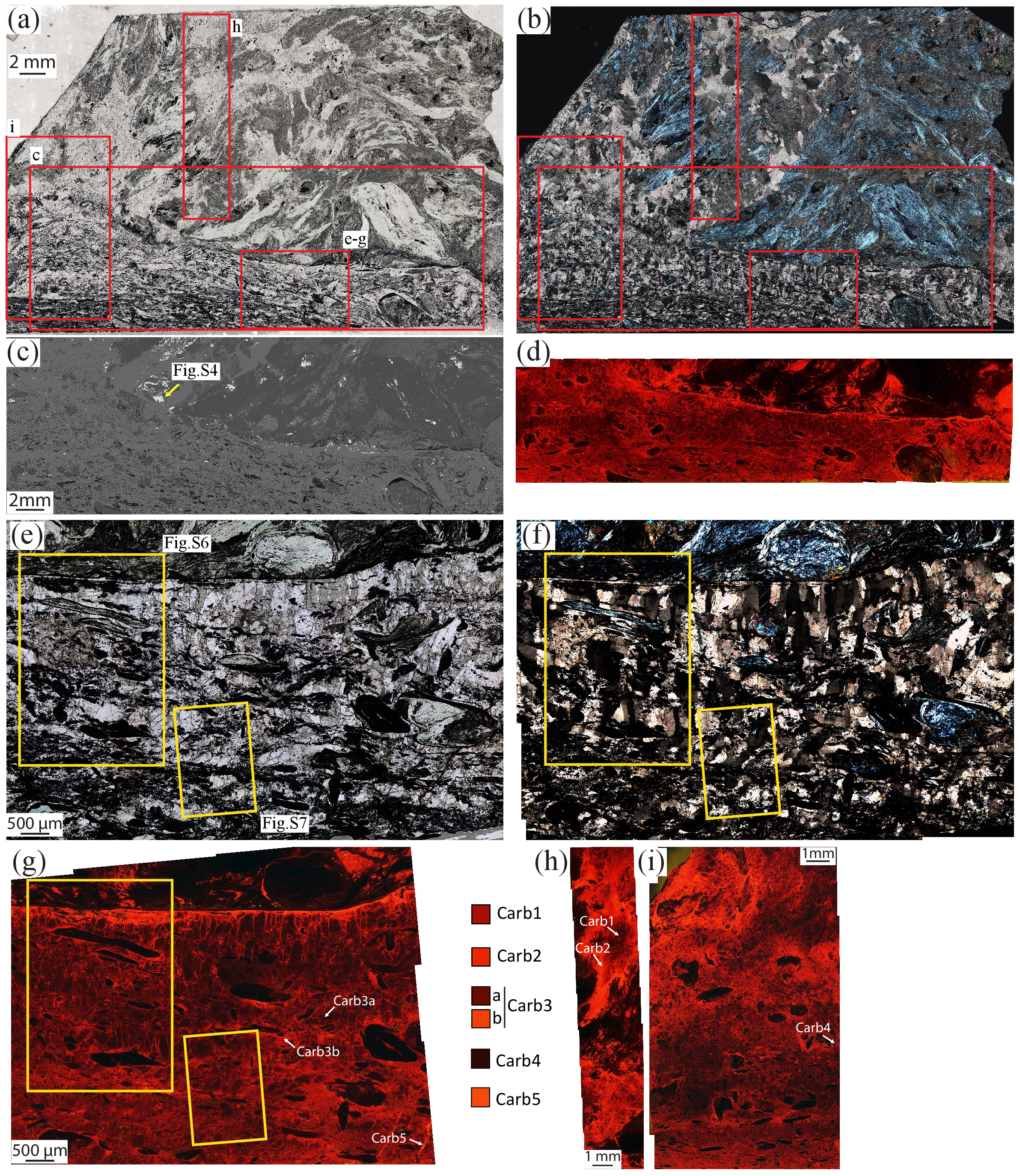
**

**Figure S5.** Microstructural features of the carbonated serpentinite (sample 5a). a-b) Thin-section optical microphotos. A composite shear zone is located at the bottom of the sample, appearing dark due to the abundant graphite and the smaller grain size of the mineral phases. The lower part of the shear zone is darker with smaller grain size (inner shear zone). Plane‐polarized light and crossed‐polarized light, respectively. c-d) Enlargement of the composite shear zone and its sharp contact with the host-rock; BSE image and CL images, respectively. e-f) Close-up showing calcite with a columnar shape in the shear zone (pseudomorphic over aragonite), serpentinite clasts and serpentine crystals elongated parallel to the shear zone, and graphite located along planes; plane‐polarized light and crossed‐polarized light, respectively. Note the graphite-rich layer at the edges of the shear zone. g-i) CL images of different structural domains with identification of the calcite generations (picture locations in a-b). g) Columnar crystals of dark Carb3a and bright Carb3b along grain boundaries and at the contact between the shear zone and the host-rock (detail in Fig. S7). h) Transect in the host-rock highlighting large Carb2 crystals with relic cores of Carb1. i) Transect through all the structural domains.

***
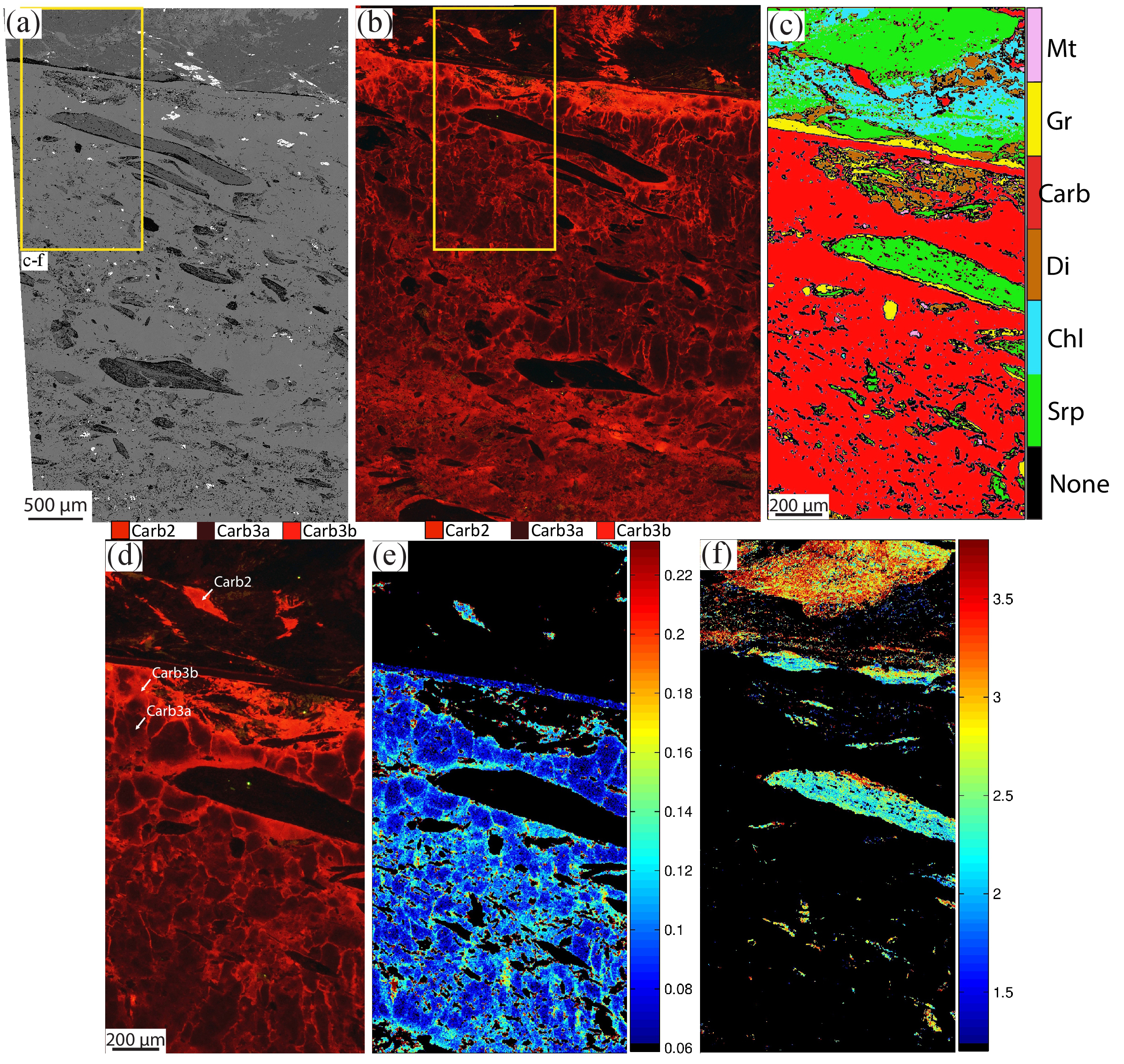
***

**Figure S6.** Details of Fig. S5e-g. a) BSE image highlighting the distribution of graphite (black) at the edges and inside serpentinite clasts (dark grey), at the edges of the shear zone, in layers inside the outer shear zone and sparse in the inner shear zone (lowermost part of the image). b) CL image highlighting the presence of frequent prismatic calcite crystals in the upper part of the image (outer shear zone) and the absence in the lower part (inner shear zone). c) X-ray map colour coded for the different mineral phases. Mt: magnetite; Gr: graphite; Carb: carbonate; Di: diopside; Chl: chlorite; Srp: serpentine. d) CL image displaying Carb3a with prismatic shape and Carb3b overgrowing it and along grain boundaries and at the contact with the host-rock. e) Standardized X‐ray map of the MgO weight %. Note the increase in MgO from Carb3a to Carb3b. f) Standardized X‐ray map of the Al_2_O_3_ weight % in serpentine, highlighting a decrease from Srp1 (in the host-rock) to Srp2 (in the shear zone).

***
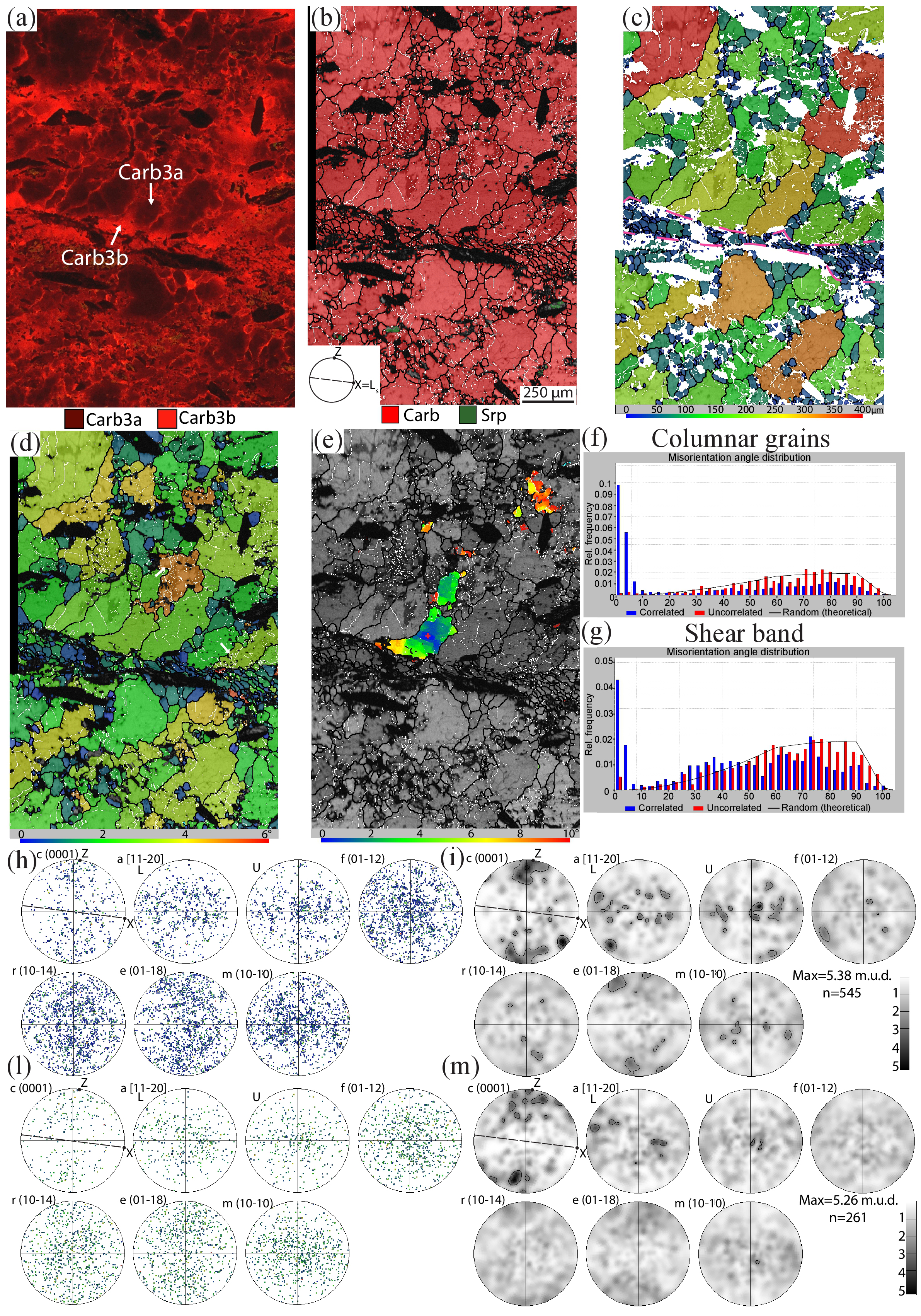
***

**Figure S7.** CL image and EBSD map 3 displaying the contact between inner (lower part) and outer shear zone (upper part), see location in Fig. S5e-g. a) CL image highlighting the textural position of the Carb3 generations. Note how Carb3b overgrows topotaxially the prismatic Carb3a crystals, especially along the shear band in a C’ orientation (compare with the EBSD maps). b) EBSD phase map. A strong grain size reduction occurs along the shear band. Columnar Carb3a grains are better preserved in the outer shear zone (upper part of the map); in the inner shear zone these are more recrystallized by subgrain rotation recrystallization. c) Calcite GS map. Columnar calcite crystals have a GS between 100 and 400 µm, calcite crystals along the shear band display an average GS of ~ 20 µm. The purple dashed line highlights the boundary of the shear band. d) Calcite GOS map. Carb3 columnar grains show GOS values between 2 and 4°, with few exceptions up to 6°; Carb3b crystals along shear band exhibit values between 1 and 2°, with one exception up to 6°. The white arrows indicate subgrains of the same size as the recrystallized grains (see text). e) TC map of Carb3 columnar grain showing increasing misorientation from core to rim. Note that the edge close to the shear band shows the highest values and corresponds to Carb3b generation in the CL image. f) and g) Histograms of distribution of misorientation angles showing the highest peaks for values <10° for correlated pairs. Columnar grains and shear band, respectively. h-m) Pole figures of the crystallographic orientation of columnar grains (h, i) and shear band (l, m). Note the strong CPO in the columnar grains, with the (0001) subparallel to the pole of the shear zone boundary. The grains in the shear band display an attenuation of the CPO. Data points are presented as one-point-per-grain. n = number of grains. Half width 10° and cluster size 5°, maximum value is given.

**
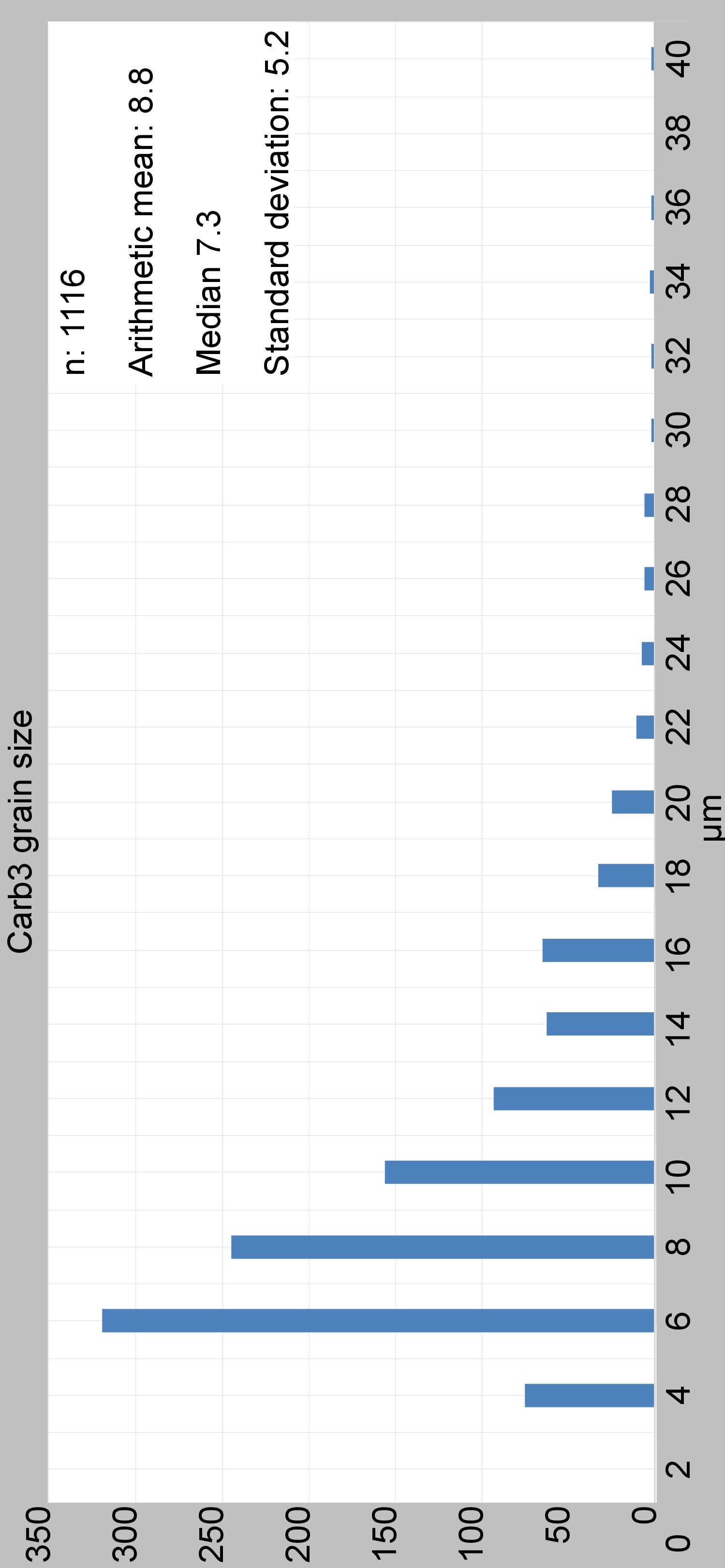
**

**Figure S8:** Grain size histogram of Carb3 grains of map2.

## References supplementary information

1. Vitale Brovarone, A. *et al.* Massive production of abiotic methane during subduction evidenced in metamorphosed ophicarbonates from the Italian Alps. *Nat. Commun.* **8**, 14134 (2017).

2. Collins, N. C. *et al.* Subduction zone metamorphic pathway for deep carbon cycling: II. Evidence from HP/UHP metabasaltic rocks and ophicarbonates. *Chem. Geol.* **412**, 132–150 (2015).

3. Giuntoli, F., Menegon, L. & Warren, C. J. Replacement reactions and deformation by dissolution and precipitation processes in amphibolites. *J. Metamorph. Geol.* **36**, 1263–1286 (2018).

4. Imon, R., Okudaira, T. & Kanagawa, K. Development of shape-and lattice-preferred orientations of amphibole grains during initial cataclastic deformation and subsequent deformation by dissolution–precipitation creep in amphibolites from the Ryoke metamorphic belt, SW Japan. *J. Struct. Geol.* **26**, 793–805 (2004).
